# A nexus of lipid and *O-*Glcnac metabolism in physiology and disease

**DOI:** 10.3389/fendo.2022.943576

**Published:** 2022-08-30

**Authors:** Amber Lockridge, John A. Hanover

**Affiliations:** Laboratory of Cell and Molecular Biology, National Institute for Diabetes and Digestive and Kidney Diseases, National Institutes of Health, Bethesda, MD, United States

**Keywords:** *O-*GlcNAc, glycosylation, hexosamine biosynthetic pathway, lipid, fatty acid, metabolism, obesity, homeostasis

## Abstract

Although traditionally considered a glucose metabolism-associated modification, the *O*-linked β-N-Acetylglucosamine (*O-*GlcNAc) regulatory system interacts extensively with lipids and is required to maintain lipid homeostasis. The enzymes of *O-*GlcNAc cycling have molecular properties consistent with those expected of broad-spectrum environmental sensors. By direct protein-protein interactions and catalytic modification, *O*-GlcNAc cycling enzymes may provide both acute and long-term adaptation to stress and other environmental stimuli such as nutrient availability. Depending on the cell type, hyperlipidemia potentiates or depresses *O-*GlcNAc levels, sometimes biphasically, through a diversity of unique mechanisms that target UDP-GlcNAc synthesis and the availability, activity and substrate selectivity of the glycosylation enzymes, *O*-GlcNAc Transferase (OGT) and *O*-GlcNAcase (OGA). At the same time, OGT activity in multiple tissues has been implicated in the homeostatic regulation of systemic lipid uptake, storage and release. Hyperlipidemic patterns of *O*-GlcNAcylation in these cells are consistent with both transient physiological adaptation and feedback uninhibited obesogenic and metabolic dysregulation. In this review, we summarize the numerous interconnections between lipid and *O-*GlcNAc metabolism. These links provide insights into how the *O-*GlcNAc regulatory system may contribute to lipid-associated diseases including obesity and metabolic syndrome.

## Introduction

Lipid homeostasis is crucial to normal physiological function. Lipids provide substrates for anabolic synthesis, act as signaling molecules or membrane property modifiers and they constitute an energy dense form of nutrient storage that can supply regulated access to cellular fuel for high demand tissues or when glucose availability is low. In the context of human evolution, the relative scarcity of high fat foods placed a premium on maximal uptake and storage of dietary lipids to ensure against future periods of nutrient scarcity. Thus, the tongue and the intestine respond to elevated lipid content by signaling to the hedonic reward system of the brain to override homeostatic control of eating (i.e. hunger and satiety) and stimulate increased consumption. Intestinal enterocytes adapt to extract more dietary fatty acids (FAs) and secrete them into the bloodstream in the form of triacylglycerol (TAG) and peripheral tissues, namely the adipose and liver, are activated to expand their cellular uptake and lipid storage capacity. On the other hand, prolonged or excessive lipid accumulation is a source of considerable cellular toxicity and stress. To mitigate these concerns, dietary lipid exposure also provides negative feedback to normalize eating patterns after a transient period of hyperlipidemia. Many cell types mitigate the risk of lipotoxicity by sequestering TAG inside phospholipid coated lipoproteins called lipid droplets (LDs). Particularly in adipocytes and hepatocytes, these can then be released back into the bloodstream during the post-absorptive state. In the state of obesity, nutrient regulation of these carefully balanced controls is diminished leading to hyperphagia irrelevant to satiety, tonically elevated systemic lipid uptake and a loss of metabolic flexibility in the liver and adipose that fuels dysregulations of lipid and glucose homeostasis, inflammation and insulin resistance that are the hallmarks of metabolic syndrome.

Metabolic flexibility is key to maintaining physiological homeostasis in response to hyperlipidemia. To make decisions about hunger vs. hedonic eating, lipid opportunity vs. toxicity and FA storage vs. release, cells need input about acute nutrient conditions but also historic nutrient availability, energy demand, storage occupancy and reserve capacity as well as cellular and tissue level stress. This information is integrated and encoded by nutrient sensitive molecules like insulin, which alters cellular behavior through receptor induced signaling, and intracellular enzymes such as AMPK and O-GlcNAc Transferase (OGT) that directly modify target proteins through phosphorylation and O-linked β-N-acetylglucosamine (*O*-GlcNAc) modification, respectively. Protein *O-*GlcNAcylation has traditionally been framed in the context of glucose metabolism, related to the derivation of its substrate, UDP-GlcNAc, from a glycolytic branchpoint. However, there is a growing appreciation of this post-translational modification (PTM) as responsive to and a regulator of all classes of nutrient metabolism, including lipids. Not only are lipids also required for the material synthesis of UDP-GlcNAc but they influence the availability and activity of all the major *O-*GlcNAc regulatory enzymes. Lipid responsive *O-*GlcNAcylation patterns show remarkable diversity in terms of tissue and context specificity, substrate targeting and temporal dynamism and are implicated in both vital physiological functions and hyperlipidemic pathology. As such, dissecting the relationship between lipids and the *O-*GlcNAc regulatory system has become increasingly relevant to understanding the physiology and pathophysiology of lipid homeostasis, with particular relevance to the onset and progression of obesity.

## Lipid influence on the *O-*GlcNAc regulatory system

Much of the classical depiction of *O*-GlcNAcylation as a nutrient-sensing modification comes from the incorporation of nutrient-derived metabolites through the various steps of the Hexosamine Biosynthetic Pathway (HBP), which produces UDP-GlcNAc. When used for post-translational *O*-GlcNAcylation, OGT catalyzes the transfer of the GlcNAc portion of this molecule onto the hydroxyl side chain of one or more serine and threonine residues inside a target protein. The *O*-GlcNAc regulatory system, as referenced in this review, is thus comprised of the enzymes that permit and facilitate HBP flux as well as the glycosylation enzymes - OGT and its GlcNAc-removing counterpart, O-GlcNAcase (OGA). Importantly, OGT, OGA and the HBP metabolites contribute to other molecular reactions as well (e.g. complex scaffolding, other glycosylation types) and so the activity and regulation of this system cannot be considered strictly synonymous with protein *O*-GlcNAcylation in all cases. Canonically, lipid influence over the *O*-GlcNAc regulatory system arises from the use of acetyl-CoA, derived from fatty acid oxidation (FAO) in many cell types, in the commitment step of UDP-GlcNAc synthesis. However, lipids also interact directly and indirectly with the expression, localization, protein binding and/or activity of all the major enzymes in this regulatory system. The partitioning of fructose-6-phosphate (F6P) between glycolysis and the HBP appears particularly relevant while the fat-sensitive targeting of OGT and OGA has been documented through a surprising diversity of mechanisms, possibly as a means towards context-specific substrate specificity. In addition, factors such as cell type, energy status, insulin signaling, lipid species and duration appear important in shaping unique relationships between hyperlipidemia and the *O*-GlcNAc regulatory system. A more precise delineation of these specific interactions is necessary, therefore, to understand the complexity and dynamism of both homeostatic and obesogenic lipid responses.

### Protein *O*-GlcNAcylation

As a nutrient sensing post-translational modification (PTM), protein *O-*GlcNAcylation is utilized to varying degrees in all cell types but is particularly active in tissues and on proteins with high relevance to metabolism (1, 2). To date, thousands of *O-*GlcNAcylated proteins have been identified (3, 4), primarily in the cytoplasm and nucleus but also in various organelles (e.g. mitochondria, LDs) (5). Proteins can have one or many OGT target sites and the modification of those sites can be acutely transient, long-lasting or semi-permanent depending on the context. The effect of *O-*GlcNAcylation can alter a protein’s stability, transcriptional, enzymatic or protein-binding activity, sub-cellular localization and/or the patterning of other PTMs (e.g. phosphorylation) (6, 7) and these changes can be inhibitory or activating, depending on the target, cell type and cellular conditions. Since its discovery, a substantial literature has developed connecting increased O-GlcNAcylation with elevated glucose levels (8, 9) but this response is also seen in other conditions such as cellular stress (10) and hyperlipidemia.

Both high fat-diet (HFD) and genetic obesity have been corelated to increased *O-*GlcNAcylation in multiple tissues, with some notable exceptions. Based on models of varying dietary fat (40-65%) and duration (1-5 months), elevated *O-*GlcNAcylation was observed in mouse liver (11, 12), aorta (13), retina (14), kidney (15), white adipose tissue (16), intestine (17) and skeletal muscle (18) and in the rat heart (19) and cerebral arteries (20). A specific increase in nuclear O-GlcNAcylation was noted in the heart and liver but not kidneys of HFD rats (21). Young male swine also had higher cardiac *O-*GlcNAcylation after 3 months on an obesogenic hypercholesterolemic diet (22). Additionally, two mouse models of non-HFD obesity (db/db and ob/ob) showed higher kidney and liver *O-*GlcNAcylation, respectively (15, 23). By contrast, hippocampal *O-*GlcNAcylation was reduced by ~30% in 6 week (wk) HFD mice compared to normal chow controls (12). This was similar to findings in male human islets in which obesity (BMI≥30) was correlated to hypo*-O-*GlcNAcylation compared to lean controls (BMI ≤ 25) (24). In some tissues, the impact of hyperlipidemia was not unidirectional but temporally dynamic and biphasic. Mouse pancreatic islets and macrophages both showed higher *O-*GlcNAcylation during the early stages of diet-induced obesity (4-6 wks) but levels fell below standard chow fed controls after 3 months in both cases (24, 25). Therefore, the effects of *in vivo* hyperlipidemia on protein *O-*GlcNAcylation, while primarily potentiating in most tissues, were depressive in the hippocampus or in islets and macrophages after prolonged exposure (**Table 1**).

**Table 1:** Effects of hyperlipidemia on protein *O-*GlcNAcylation in different tissues.

Tissue	Model	Duration	*O-*GlcNAc	HBP	OGT	OGA
Stomach	[Sat] LCFA *(h)*	3hr	↑	↑GFAT	↑	–
Intestine (Colon)	HFD *(m)*	8w	↑	–	–	–
Adipose	HFD *(m)*	4w	↑	–	–	–
Skeletal Muscle	[Sat] LCFA *(h, r)*	15-20hr	↑	–	–	–
	HFD *(m, r, sw)*	3d-16w	↑	↑ GFAT		↓
Heart	[US] LCFA *(r)*	3d	↑	↑GFAT	–	–
	HFD	*(m, r)* 7-22w	↑	↑GFAT	↑	↓
		*(sw)* 7-12w	↑	↓ UDP-GlcNAc	↔	
Liver	[Sat, US] LCFA *(h, f)*	12-24hr	↑	–	↑	↔ or ↓
	HFD *(m, r, f)*	3d-12w	↑	–	↑	↓
	Obesity *(m)*	3-14w	↑	↔UDP-GlcNAc ↔GFAT	–	–
Islets/ β-cells	[Sat] LCFA *(h)*	2hr	BMI≥30 ↑	–	–	–
			BMI≤25 ↔			
	HFD *(m)*	6w	↑	–	M ↑	M↑
					F ↔	F ↔
		18w	↓		↓	–
	Obesity *(h)*	BMI≥30	F ↔	–	F ↔	–
			M ↓		M ↓	
Macrophage	[Sat] LCFA *(m)*	2-24hr	↑	–	↔	↔
	HFD *(m)*	1-4w	↔ or ↑	–	–	–
		12w	↓			
Brain	[Sat] LCFA *(h)*	24hr	↓	–	–	–
	HFD *(m, r)*	6w	↓	↔ GFAT ↑pGFAT1	↓pTyr	↔

Summary data are shown for studies reporting changes in *O-*GlcNAcylation in metabolically important tissues (for full data and references, see **Supplemental Table 1**). Elements supporting a positive correlation between hyperlipidemia and *O-*GlcNAcylation are coded in blue and negative relationships in red. Models include *in vitro* long-chain fatty acids (LCFA), high fat diet (HFD) and obesity (human or genetic driven obesity). The species (cells or tissues) included in the model summary are indicated as human (*h*), mouse (*m*), rat (*r*), swine (*sw*) and fish (*f*). For LCFAs, the use of saturated acids ([Sat] = palmitate) or unsaturated acids ([US] = oleate) is described. Duration describes the range of exposure times used (LCFA, HFD) or the age/BMI of the subjects (animal/human obesity) in terms of hours (hr), days (d), or weeks (w). Sexually dimorphic findings are labeled as male (M) or female (F). Findings on *O-*GlcNAc regulatory elements (HBP enzyme GFAT and output UDP-GlcNAc, OGT, OGA) are only included for studies that also reported *O-*GlcNAcylation in the same model. A dash indicates no reported data.

Similar to the obesity models, *in vitro* exposure to lipids, especially long-chain fatty acids (LCFA), triggered a rise in *O-*GlcNAcylation in all but a select few cell types. Palmitate (PA), which is the most abundant saturated LCFA in most HFDs, increased total protein *O-*GlcNAcylation in immortalized cell lines from mouse and human liver (26, 27), rat retina (14) and two human-derived gastric cancer cell lines (28). PA also stimulated *O-*GlcNAcylation in primary isolated cells including rat L6 myotubes (29), kidney mesangial cells (30), mouse bone-marrow-derived macrophages (25) and human obese donor male islets (24). Notably, this latter observation was not seen for the lean donor cells, suggesting that pre-existing obesity may have sensitized the islets to PA’s acute effects on the *O-*GlcNAc regulatory system (24). By contrast, PA led to a 50% decrease in the *O-*GlcNAcylation of SHSY-5Y human neuroblastoma cells, mimicking the similar effect of HFD on the hippocampus (12). Oleate (OA), an endogenously abundant unsaturated LCFA, was reported to drive protein *O-*GlcNAcylation in primary hepatocytes from fish (31) and rat neonatal cardiomyocytes (32). Importantly, LCFA concentrations in these experiments, 100-600 uM PA or 400-800 uM OA, were within the physiological range for human plasma (33) although the durations of exposure varied widely by experiment, from 2 to 48 hours (see **Supplementary Table S1** for study details). Beyond LCFAs, increased cellular *O-*GlcNAcylation was documented in response to multi-lipid enriched media for mouse oocytes (34), a biologically active ceramide analog (Cer6) in rat retinal cells (14) and short chain fatty acids in intestinal epithelial cells (17). Both the *in vivo* and *in vitro* outcomes are largely aligned with the perception of *O-*GlcNAcylation as a general marker of nutrient excess in most tissues. It is worth noting, however, that relatively few studies reported multiple timepoints, which is likely important in distinguishing the physiological and pathophysiological relevance of outcome.

A notable exception to the predominant pattern of hyperlipidemic hyper-*O-*GlcNAcylation was the lipid-stimulated depression of *O-*GlcNAcylation in the mouse hippocampus and human neuroblastoma cells. The rationale for this contrary effect is not definitive but several interesting hypotheses arise. Most brain cells, including neurons, have a low capacity for FAO, relying almost exclusively on glucose oxidation or sometimes ketones, to fuel cellular activity [see (35)]. At the same time, glucose-driven *O-*GlcNAcylation is neuroprotective against multiple cognitive disorders [for review (36)]. Lipotoxic insulin resistance in the brain disrupts glucose metabolism and has been proposed as a causal link between obesity and Alzheimer’s disease (12). Therefore, the rationale and/or mechanisms for lipid sensing and lipid modulation of protein *O*-GlcNAcylation may be different in these cells compared to others. For example, in hyper-*O*-GlcNAcylated cell types, lipids appear to partition F6P towards the HBP at the expense of glycolysis (see “UDP-GlcNAc Synthesis” for details). In cells that cannot alternately utilize fatty acids as a fuel source, this would come with considerable energetic costs, especially in electrically active neurons. To that point, neural hypo-*O*-GlcNAcylation was dependent on the low energy sensor AMPK, both in this model (12) and in glucose-deprived neuroblastoma cells (37). In addition to preserving glycolytic substrate, reducing neural *O*-GlcNAcylation may stimulate LD growth as a method to sequester toxic lipid excess. It was recently shown that decreased *O*-GlcNAcylation of the TATA box binding protein (TBP) shifts its influence over the transcription pre-initiation complex proteins, leading to broadly increased lipogenic gene expression associated with rat hippocampal LD accumulation (38). Hyperlipidemic *O-*GlcNAcylation patterns have not yet been characterized in areas of the brain more directly involved in lipid sensing and nutrient metabolism (e.g. the hypothalamus). However, fasting represents an endogenous state of low energy with high circulating free FAs (FFAs) and has been shown to increase *O-*GlcNAcylation in FAO*-*capable AgRP neurons of the hypothalamus while the opposite occurs in glucose-utilizing POMC neurons (discussed in section OGT activity and dietary lipid uptake). Therefore, the metabolic flexibility and lipotoxic vulnerability of a given cell type, even more than its anatomical location, may be a significant contributor to its *O-*GlcNAc lipid response.

Both *in vivo* and *in vitro* hyperlipidemia models demonstrate that lipids increase protein *O-*GlcNAcylation in most cell types. Macrophages and islet cells, however, showed a biphasic relationship between the duration of lipid exposure and *O-*GlcNAcylation, first potentiating and then inhibitory. This is consistent with the dynamism of their functional adaptation to progressive obesity, discussed later in this review [but see also (39, 40)]. Furthermore, hyperlipidemia suppressed *O-*GlcNAcylation in some brain cells, possibly related to the unique neuroprotective role of this modification and/or an enhanced sensitivity to lipotoxicity in the absence of lipid oxidation as a viable fuel source.

### UDP-GlcNAc synthesis

**Figure 1:**
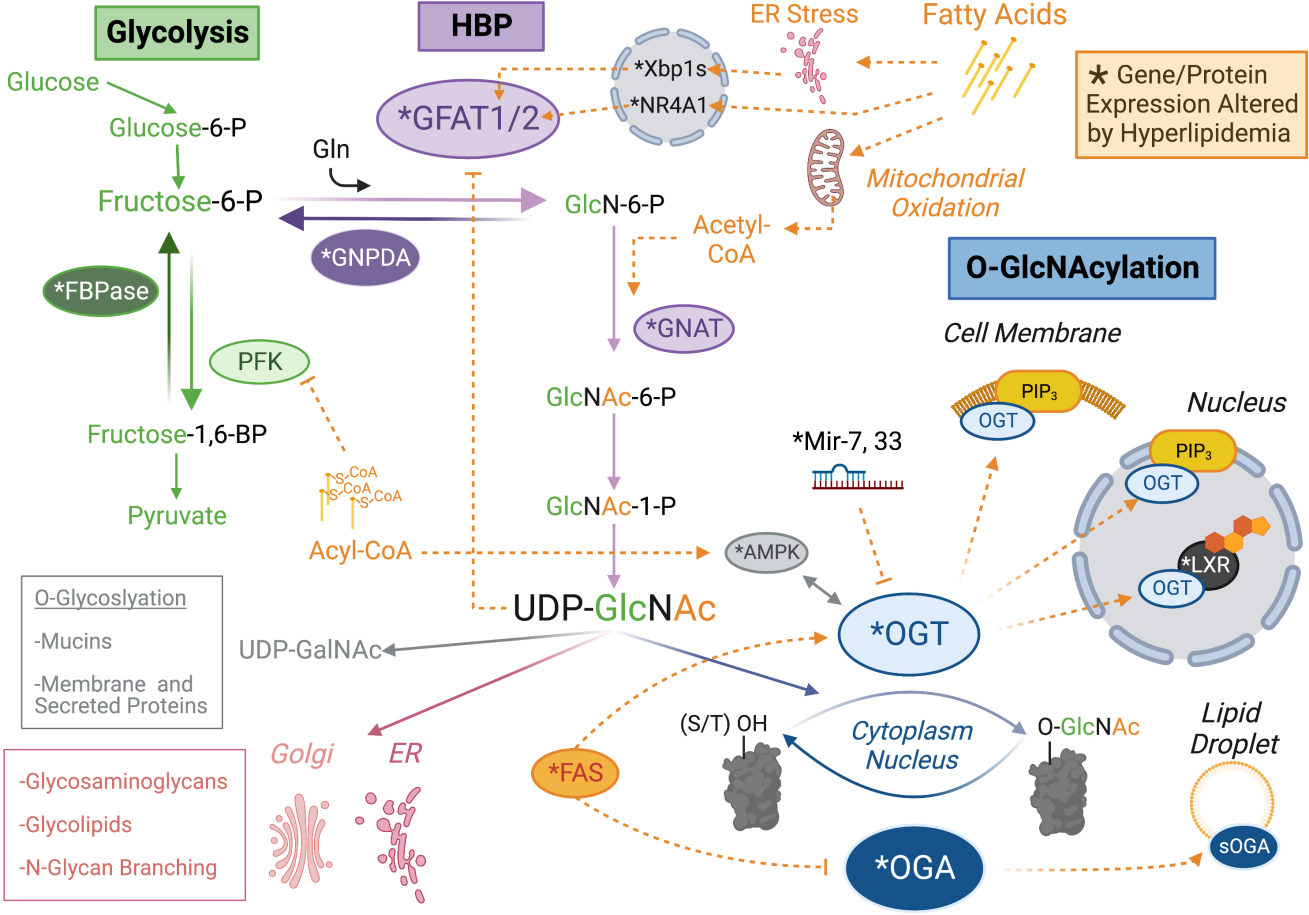
Lipid Control Over the *O-*GlcNAc Regulatory System. Protein *O-*GlcNAcylation (blue) is regulated by the activity or localization of the glycoslylation enzymes, *O-*GlcNAc Transferase (OGT) and *O-*GlcNAcase (OGA) through direct binding to lipid species (e.g. PIP3) or downstream of lipid sensitive proteins and miRNAs. The substrate for OGT is UDP-GlcNAc, which is synthesized through an offshoot of glycolysis (green) called the hexosamine biosynthetic pathway (HBP, purple). Lipid regulation of the HBP seems particularly focused on expression of the rate limiting enzyme GFAT and control over the metabolic fate of fructose-6-phosphate (P), which represents the divergence point between glycolysis and the HBP. Proteins (or microRNA) with an altered expression profile in obesity are indicated by asterisk (see main text for details). Orange dashed lines indicate agonistic (➔) or inhibitory (–|) relationships between lipid metabolites (e.g. Acyl-CoA) or lipid regulated species (e.g. Fatty Acid Synthase, FAS). It is important to note that UDP-GlcNAc has multiple utilizations in the cell, which might also be influenced by the regulatory relationship indicated. Post-translational modifications are not included for the sake of visual clarity. Created with BioRender.com.

One method for lipids to impact cellular *O*-GlcNAcylation levels is through the enzymes that regulate UDP-GlcNAc synthesis. The production of UDP-GlcNAc through the HBP starts with F6P, primarily derived from the early steps of glycolysis. If phosphorylated by phosphofructokinase 1 (PFK-1), F6P can continue down the glycolytic path as fructose-1,6-bisphosphate (FBP) or it can be diverted towards the HBP by the rate-limiting enzyme glucosamine-F6P-aminotransferase (GFAT). Conversely, F6P can be pulled back from either pathway through the reversal of these reactions, mediated by the enzymes FBPase-1 and glucosamine-6-phosphate deaminase (GNPDA), respectively. Thus, PFK/FBPase and GFAT/GNPDA represent critical choice points over the metabolic fate of F6P, which ultimately dictates the proportion of glucose dedicated to UDP-GlcNAc synthesis [see (41–43)]. Importantly, GFAT is expressed in two paralogous forms as *Gfpt1*/GFAT1 and *Gfpt2*/GFAT2. Despite having a similar role in the HBP and ~75% sequence conservation, these two proteins exhibit independent regulatory, developmental and tissue expression patterns (14, 44–47) suggesting unique but as yet poorly distinguished physiological roles. GFAT transfers an amide group from the amino acid glutamine (Gln) to convert F6P to glucosamine-6-phosphate (GlcN-6-P). Subsequently, GlcN-6-P acetyltransferase (GNAT) uses acetyl-CoA as a substrate to form N-acetylglucosamine-6-phosphate (GlcNAc-6-P), committing pathway flux in the forward direction. In the final steps of the HBP, UTP hydrolysis fuels the formation of uridine diphosphate-N-acetylglucosamine (UDP-GlcNAc). UDP-GlcNAc, and its epimers UDP-GalNAc and ManNAc, contribute to multiple pathways (e.g. mucin-like *O-*glycosylation, glycosaminoglycan synthesis), but UDP-GlcNAc availability is most sensitively linked to OGT-mediated *O-*GlcNAcylation (48). This pathway, and its putative regulation by lipids, are presented graphically as a component of **Figure 1**.

The reported outcomes of hyperlipidemia on HBP activity have been varied, dependent on the experimental approach. UDP-GlcNAc was elevated in the skeletal muscle of ob/ob and KKAy genetically obese mice (49, 50) and in WT mice after 3 days HFD (51) or following *in vivo* lipid infusion in rats (52). Despite this, a later study found no effect of intralipid infusion on muscle HBP products (53) and UDP-GlcNAc levels were unchanged in the skeletal muscle of Zucker Diabetic Fatty rats compared to lean controls (54). Furthermore, UDP-GlcNAc was normal in the livers of the same ob/ob and KKAy mice with elevated muscle levels (49, 50). This last finding may be attributable, in part, to the higher Km of GFAT for F6P in rodent skeletal muscle compared to other tissue types (~2.4 vs. <0.5 mM), meaning GFAT could have been saturated in the liver (49). However, UDP-GlcNAc was decreased in the HFD swine heart simultaneous with a measure of increased *O-*GlcNAcylation (22), illustrating the fact that the static pool of UDP-GlcNAc is an imperfect measure of either HBP flux or HBP-driven *O-*GlcNAcylation. UDP-GlcNAc is rapidly depleted as a substrate and feeds back to inhibit GFAT, such that the impact of transient elevations on OGT activity may be missed (33, 53). In fact, a general limitation of the field is that HBP flux, defined as the rate of molecular turnover through this pathway, is not well understood.

As an alternate approach, several studies have focused on lipid regulation of the HBP enzymes themselves. *Gfat* mRNA was increased in HFD mouse retina (14) and porcine muscle (55) while protein levels were up in the HFD mouse aorta (56) and higher activity was directly measured in ob/ob mouse muscle and fat tissue (49). Similarly, human BMI was positively correlated to GFAT activity in non-diabetic human muscle cells (57). GFAT’s counter-regulatory enzyme partner, GNPDA, has also been implicated in human obesity (58) and was transcriptionally downregulated in the rat hypothalamus after 6 wks of HFD (59). The exception to these findings was an increase in inhibitory phosphorylation of GFAT1 in the mouse hippocampus, but this was consistent with the decrease in protein *O-*GlcNAcylation observed in that tissue after HFD (12). *In vitro*, human-derived myocytes showed increased GFAT protein and/or mRNA after a 20-hour exposure to saturated LCFAs (PA, stearate) while unsaturated LCFAs (OA, linoleate, palmitoylate) had little or no effect in the same study (33). However, a longer 3-day OA incubation did elevate GFAT protein in a separate investigation on rat neonatal cardiomyocytes (32). Alltogether, the evidence suggests that GFAT activity is closely correlated to the outcome of hyperlipidemia on *O-*GlcNAcylation, presumably through its effects on the availability of UDP-GlcNAc.

However, several studies suggest that GFAT1 and GFAT2 are differentially impacted by lipid exposure. Dai *et al.* reported that PA, Cer6 and HFD all increased rodent retinal *Gfpt2* transcription, as well as protein *O-*GlcNAcylation, but *Gfpt1* mRNA was increased, decreased or unchanged by each respective condition (14). In mouse oocytes, multi-lipid media supplementation triggered an increase in *Gfpt1* after 8 hours, falling to baseline by 16 hours whereas *Gfpt2* was only observed to decrease and at the latter timepoint (34). Some increases in GFAT transcription might be explained by lipotoxicity. ER stress, a well-characterized downstream effect of lipid accumulation (60), triggers the splicing activation of Xbp1, which can then act in the nucleus as a *Gfpt1* transcription factor (61), driving up UDP-GlcNAc synthesis (61, 62). In addition, both HFD and *in vitro* lipids (PA, Cer6) increased the expression of lipotoxicity sensor and nuclear receptor protein NR4A1 leading to the specific upregulation of GFAT2, but not GFAT1, in the rodent retina (14). Differential nutrient regulation of the GFAT paralogs was also observed in *Drosophila* wherein dietary GlcN-6-P, the product of GFAT HBP activity, rescues lethality in *gfat2* but not *gfat1* knockout (KO) flies (63). Nevertheless, ectopic overexpression of either protein is sufficient to rescue loss of the other (47), suggesting that the nutrient access and/or responsivity of *gfat1* and *gfat2* expressing cells may be meaningfully distinct. Indeed, a recent study in WT flies confirms that of the two genes, only *gfat2* expression is correlated with circadian eating patterns (64). By contrast, *gfat1* may be more sensitive to lipid-induced stress, which is consistent with its proposed role in mediating the depressive effects of hyperlipidemia on neural *O*-GlcNAcylation. Unfortunately, much of the available data on lipids/obesity and GFAT does not differentiate between the paralogous forms, which may be a key detail underlying the diversity of hyperlipidemic outcomes in different cell types or conditions.

The availability of F6P for GFAT, which is rate-limiting in mammalian cells (49), is another target for lipid regulation of HBP flux. The enzyme PFK, which moves F6P towards glycolysis, is inhibited by LCFA-CoAs, an activated form of LCFA important in cellular lipid metabolism. LCFA-CoA binding to purified rabbit muscle PFK-1 *in vitro* exposed a tryptic cleavage site for the enzyme (65). Palmitoyl-CoA also triggered the inhibitory acylation of PFK-1 cysteine residues near the enzyme’s nucleotide-binding site. Furthermore, cytoplasmic citrate, which accumulates in response to high FAO activity, is also a well-known allosteric inhibitor of PFK [for review (66)]. Since PFK inhibition blocks glycolysis, this could increase F6P availability for GFAT-mediated HBP flux. Towards a similar outcome, LCFAs potentiate FBPase, which would pull F6P back from glycolysis by dephosphorylating FBP. PA or OA-supplemented media increased gene expression of both the liver and muscle isoforms of FBPase, as well as the HBP enzyme GNAT, in pancreatic β-cell line Min6 (67). These findings are consistent with a hypothesis of competitive glycolytic and HBP flux in response to normoglycemic but hyperlipidemic conditions.

In sum, HBP throughput appears to be a common target of lipid regulation with particular emphasis on GFAT and enzymes that direct the metabolic fate of F6P. In most cases, lipid exposure potentiated HBP-promoting proteins (GFAT, GNAT, FBPase-1) and inhibited HBP-detracting proteins (GNPDA, PFK-1), consistent with models of hyper-*O*-GlcNAcylation. However, the GFAT1 and GFAT2 paralogs appear to be independently regulated by lipids, dependent in part on cell type, lipid species and duration of exposure, energy status and cell stress. In particular, AMPK-dependent inhibition of GFAT1 was associated with the lipid depression of *O*-GlcNAcylation in some brain cells.

### *O-*GlcNAc enzymes

In addition to UDP-GlcNAc synthesis, lipids may impact protein *O*-GlcNAcylation through the GlcNAc attachment and detachment enzymes, OGT and OGA. Although this reaction is catalyzed by only a single pair of enzymes, the capacity for multiple regulatory levels (i.e. expression, function, protein binding and/or localization) provides opportunities for substrate or context-specificity [for review (68)]. The ordered bi-bi enzyme mechanism utilized by OGT requires that UDP-GlcNAc bind prior to peptide binding (69). Both OGT and OGA act in a distributive rather than processive fashion suggesting that multiple cycles of binding and dissociation reactions are required (70). Moreover, both enzymes express multiple splice isoforms, including a shortened version of OGA (sOGA), which lacks the histone acetyltransferase domain of the longer and predominantly active protein and shows selective localization to LDs (71) and mitochondria (72) where lipid metabolic processes are highly active. The three OGT isoforms differ in localization but also in the length of their tetratricopeptide repeat (TPR) domains, which mediate non-catalytic protein binding, interactions that facilitate glycosyltransferase substrate selectivity as well as OGT’s non-enzymatic structural functions (e.g (73).). Furthermore, both OGT and OGA are subject to post-translational modifications, including *O-*GlcNAcylation and phosphorylation, that impact their function and protein interactions (74). Lipids appear to target OGT and OGA at multiple levels within this mechanistic diversity. It is important to acknowledge, however, that many counter-regulatory mechanisms exist to keep *O*-GlcNAcylation levels within a physiologically optimal range [see (75, 76)], such that effects on any particular element of the system may indirectly influence other elements.

In obesity models, OGT and/or OGA protein were not always altered but when they were, it was generally consistent with the change in protein *O-*GlcNAcylation. Among tissues with HFD hyper-*O-*GlcNAcylation – OGT was elevated in the mouse liver and heart (11, 27, 56) while OGA was suppressed in skeletal muscle and heart (18, 22, 56). In islets, depressed *O-*GlcNAcylation was accompanied by reduced OGT levels (24). Interestingly, higher islet *O-*GlcNAcylation after a moderate HFD period, was associated with either no change in OGT and OGA (females) or simultaneous unidirectional change (males), indicating an increased *O-*GlcNAc cycling rate. Contrary to islets, *O-*GlcNAcylation loss in the mouse HFD hippocampus was correlated with normal OGT protein and OGA activity but OGT PTMs were altered (decreased *O-*GlcNAcylation and tyrosine phosphorylation, increased Ser/Thr phosphorylation) (12). Recently, LCFA-CoAs were found to allosterically activate AMPK (77), a Ser/Thr kinase with high expression in hippocampal neurons (78) and increased activation in the HFD hippocampus (12). OGT phosphorylation by AMPK shifts its subcellular localization and substrate specificity in myotubes (79) and it would be interesting to see whether it has a similar role in the brain. Only one study reported on GFAT, OGT and OGA together, finding that HFD increased the first two and decreased the latter in the mouse aorta (56), working collectively to elevate *O-*GlcNAcylation by increasing substrate and transferase activity while decreasing *O-*GlcNAc removal.

Data from *in vitro* experiments is more preliminary but suggests that lipid regulation of OGT and OGA is less direct than for F6P and HBP enzymes. Two studies found increased *OGT/ogt* mRNA following LCFA exposures – with PA in human gastric cancer cells (28) or OA in primary hepatocytes from yellow croaker fish (31). However, PA in mouse liver and macrophage cells had no effect on transcription of *Ogt* or *Oga* (25, 26). A hyperlipidemia responsive *Ogt* transcription factor has not been definitively identified. Nevertheless, two microRNAs that are highly involved in lipid metabolism target OGT post-transcriptionally – Mir-7 [confirmed (80, 81)] and Mir-33 [putative (82)]. Mir-33 is co-expressed with the lipid transcription factor, SREBP2, in response to low cholesterol levels (83). Mir7 expression is bidirectionally regulated by *in vitro* LCFAs (84) and HFD feeding (85), possibly downstream of another lipid transcription factor, PPARα (86). In islets, Mir-7a expression was reduced after 5 wks HFD but increasingly expressed beyond that timepoint (85), consistent with the timeframe of elevated and then repressed OGT expression found in a separate HFD islet study (24). On the other hand, fatty acid synthase (FAS), which is the rate limiting enzyme of *de novo* lipogenesis, and also binds to and influences both OGT (87) and OGA (23) in an *O-*GlcNAc elevating direction. Pharmacological FAS inhibition dose-dependently suppressed HepG2 OGT protein levels (23). By contrast, FAS-bound OGA showed an ~85% decrease in activity compared to unbound OGA in a human-derived bone cancer cell line (88). Murine obesity increases the interaction between FAS and OGT in the liver, correlated to higher protein *O-*GlcNAcylation and lipogenic activity in that tissue (87). These connections provide plausible paths by which hyperlipidemia may influence the protein expression or activity of OGT and OGA.

As a means to guide substrate targeting, lipids have also been shown to influence the subcellular localization of the *O-*GlcNAc enzymes. OGT binds to phosphatidylinositol phosphate (PIP) and its derivatives, showing high specificity for PIP3 in vitro (89, 90), although a definitive binding domain has yet to be identified (91). PIP3 binding does not alter OGT enzymatic activity but it was reported to recruit OGT to the plasma membrane in response to serum or insulin in Cos7 kidney-derived cells (89) and HepG2 human liver cells (92). Following high-glucose culture, OGT moved to the nuclear membrane of MIN6 β-cells and co-immunoprecipitated with PIP3 in nuclear extracts (90). In all cases, the triggering stimulus was pharmacologically identified to be PI3 kinase activation, which converts membrane bound PIP2 to PIP3. Accordingly, loss of PTEN, the enzyme that dephosphorylates PIP3, was correlated to decreased OGT protein in the mouse liver (23). In addition to PIP3, OGT binds to oxysterol-activated liver-X receptor (LXR) and co-localizes with it in the nucleus of transfected Huh7 human hepatoma cells (93). LXR loss in mice does not alter *OGA* mRNA or OGT protein level but does selectively reduce nuclear *O-*GlcNAcylation (93). Protein expression of the LXRα paralog is enhanced by HFD in the rat liver (94–96) and in HepG2 cells by 24 hours *in vitro* PA (96). Less data is available on OGA localization patterns but acute OA potentiates the activity of the sOGA isoform and stimulates its accumulation on LD membranes in HeLa cells and 3T3-L1 pre-adipocytes (71) through an unknown mechanism. The extent and physiological relevance of these localization shifts in the endogenous cellular environment, whether enzymatic or non-catalytic, remain largely unknown but suggest an intriguing complexity to lipid regulation of OGT and OGA that may not be apparent in common endpoint measures of total protein level or activity.

Changes in OGT and OGA were sometimes but not always apparent in models of lipid altered *O-*GlcNAcylation. Plausible mechanisms linking lipids to OGT/OGA activity include lipid sensitive micoRNA transcription and interactions with nutrient/energy-sensing proteins that impact protein stability, enzymatic activity, PTM patterns and subcellular localization. As a general conclusion, lipids not only impact overall *O*-GlcNAc tone but are capable of diverse mechanistic influences that tune elements of the *O*-GlcNAc regulatory system in different ways. Simultaneously, hyperlipidemia can be expected to modify the ambient cellular environment, incorporating tissue and context dependent cues such as nutrient history, energy status and stress to further direct *O*-GlcNAcylation patterns towards specific outcomes.

## OGT activity and dietary lipid uptake

**Figure 2:**
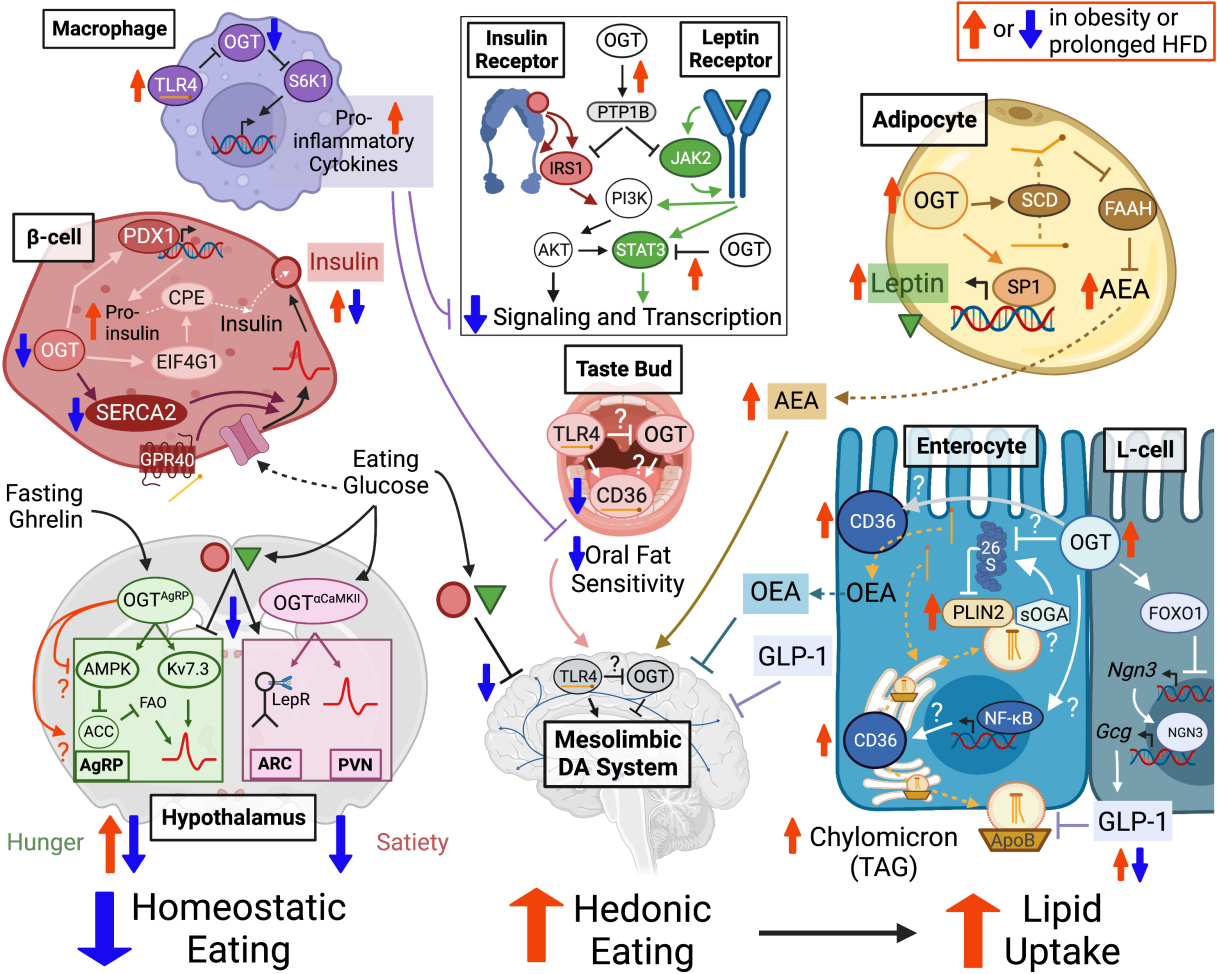
OGT supports neural activity in hypothalamic nuclei that regulate homeostatic eating drives including hunger-stimulating AgRP neurons and satiety-promoting neurons in the ARC and PVN. OGT in β-cells and adipocytes is important for the production of insulin and leptin hormones, which promote satiety and inhibit mesolimbic dopamine circuits that control hedonic eating motivation towards high fat foods. OGT activity facilitates transient lipid hyperphagia through the stabilization of adipocyte endocannabinoid AEA but may also contribute to counter-balancing feedback through oral fat sensitivity and orexigenic intestinal hormone secretion (i.e. OEA, GLP-1). The *O-*GlcNAc potentiated and lipid binding proteins CD36 and PLIN2 are promising candidates for regulating systemic lipid uptake through lingual fat detection and/or enterocyte extraction of dietary fatty acids into circulatory particles of chylomicron TAG. These mechanisms support a physiological eating pattern driven by homeostatic motivations punctuated by transient hedonic overconsumption of high palatability foods. However, OGT is also implicated in mechanisms underlying obesogenic pathophysiology. Prolonged hyperlipidemia decreases macrophage *O-*GlcNAcylation to disinhibit TL4-mediated pro-inflammatory cytokine secretion that can blunt insulin and leptin receptor sensitivity as could OGT targeting of receptor signaling modulators PTP1B and STAT3 in multiple cell types. Persistent high fat exposure decreases *O-*GlcNAcylation in β-cells but potentiates it in adipocytes and intestinal epithelial cells, potentially contributing to the high basal but impaired nutrient regulation of mature insulin and leptin secretion, elevated AEA levels and increased dietary lipid extraction that characterize a shift in obesity away from homeostasis and towards the chronic overconsumption of fat. OGT in this diagram represents the output of OGT activity (e.g. *O-*GlcNAcylation or protein interactions), whether driven by UDP-GlcNAc synthesis, OGA or OGT itself. “?” indicates a regulatory mechanism that has been demonstrated in other cell types but only hypothesized in the current setting. Dashed arrows show movement and solid arrows show effect, whether potentiating (–>) or inhibitory (–|). A double arrow combination (1 up, 1 down) indicates an increase in tonic tone with a depression in nutrient-responsive potentiation. Created with BioRender.com. Abbreviations: Agouti-related protein (AgRP) expressing, Arcuate Nucleus (ARC), Paraventricular Nucleus (PVN), O-GlcNAc Transferase (OGT), Dopamine (DA), N-arachidonoylethanolamine (AEA), Oleoylethanolamide (OEA), Cluster of Differentiation 36 (CD36), Triacylglycerol (TAG), Protein Tyrosine Phosphatase 1B (PTP1B), short-isoform O-GlcNAcase (sOGA), Perilipin 2 (PLIN2), Toll-Like Receptor 4 (TLR4).

As described in section **Lipid influence on the O-GlcNAc regulatory system**, protein *O-*GlcNAcylation is regulated by hyperlipidemia and obesity. However, this relationship is bidirectional. Many of the factors which contribute to circulatory and tissue hyperlipidemia are controlled, directly or indirectly, by *O-*GlcNAcylated proteins. This includes influence over total food consumption and specific lipid uptake, at the level of fat preference and intestinal absorption. The homeostatic drive to eat is controlled primarily in the hypothalamus by the balance between orexigenic (hunger) and anorectic (satiety) signaling. However, eating can also be stimulated by appetitive drives towards palatable foods (e.g. high fat, high sugar) that are regulated by the brain’s mesolimbic reward circuitry. Activity in these regions is responsive to the local cellular environment but also to secreted signals from the body about historic and acute conditions of nutrient availability, energy demand and dietary content. Beyond food quantity and content, actual lipid uptake depends on the rate and capacity of intestinal absorption. Ingested lipids are primarily broken down into FFAs to be packaged by enterocytes into TAG or other storage lipids, at the core of chylomicron particles that are secreted into the bloodstream. Obesity is associated with a loss of acute nutrient signaling in the brain, intestine and peripheral metabolic tissues combined with tonic physiological shifts that stimulate overconsumption, dietary lipid preference and enhanced uptake. OGT appears necessary for the nutrient-sensitive regulation of normal eating patterns but *O-*GlcNAcylation is also implicated in mechanisms underlying these obesogenic phenotypes. **Figure 2** summarizes the known and suspected roles of the *O-*GlcNAc regulatory system in dietary lipid uptake under physiological and obese conditions, as detailed below.

### Hunger

Hunger is stimulated by the activity of orexigenic NPY/AgRP neurons in the hypothalamic arcuate (ARC) nucleus in response to fasting conditions. Progressively during the pre-prandial period, gastric ghrelin (97) and adipocyte-secreted uridine and FFAs (98) are released into the bloodstream where they can be taken up by cells in the hypothalamus. Ghrelin and purine receptor activation, the latter from uridine-driven UDP synthesis, potentiate NPY/AgRP neural activity (99, 100). AgRP activity subsequently inhibits anorectic signaling in the brain’s satiety centers, namely ARC POMC neurons and the paraventricular nucleus (PVN), as well as influencing peripheral glucose metabolism [for review (101)]. ARC neurons are also capable of direct nutrient sensing of glucose and fatty acids [see (102)]. Fasting increases circulatory FFAs in the absence of elevated glucose/insulin (see section O-GlcNAcylation in lipid storage and release ). Moderate LCFA uptake under these conditions stimulates AgRP neural activity by fueling FAO-dependent ATP production. This is facilitated by ghrelin receptor signaling, which mitigates negative feedback on the FAO rate from lipogenesis and oxidative stress (103). At the same time, basal glucose levels in the fasting state limit energetic support for anorectic POMC neurons, which derive ATP through glucose oxidation (104). Thus, a suppression in satiety signaling works with increasing hunger signals to provide a building trigger to eat.

Obesity decreases fasting-stimulated hunger while maintaining anti-satiety signaling during all nutrient states. In mice, obesity abrogates the fasting-induced rise in serum uridine (105). By contrast, post-prandial uridine is elevated in obese humans (106) and hypothalamic UDP is increased in obese mice (100). Similarly, fasting levels of ghrelin are reduced in human obesity but so is the magnitude of meal-induced ghrelin suppression (101, 107). Ghrelin resistance develops in AgRP neurons, depressing their stimulated activation, but inhibition of the PVN is maintained (101). While short-term high fat feeding in rats enhances AgRP FAO capacity (108), contributing to HFD hyperphagia (see “Hedonic Eating”) long-term HFD desensitizes AgRP neurons to both fasting and dietary fat exposure (109) and abrogates the glucose sensitivity of POMC neurons (110) in mice. These changes demonstrate the importance of AgRP activity in lipid-sensitive physiological responses and show how chronic hyperlipidemia uncouples orexigenic signaling from its appropriate nutrient context.

Protein *O-*GlcNAcylation has been implicated in AgRP excitability and ghrelin sensitivity. Both fasting and ghrelin increase OGT and *O-*GlcNAcylation levels in ARC AgRP neurons, specifically including the Kv7.3 potassium channel (111). Kv7.3 potentiates neural firing rate by helping to re-polarize the cell after an action potential, which shortens the inhibitory period before the next depolarization. Mutagenic loss of the primary Kv7.3 *O-*GlcNAc site decreased its activity at depolarized potentials, correlating to a reduction in AgRP excitation rate in *AgRP-Ogt* KO mice. Furthermore, these mice were insensitive to ghrelin or fasting induced suppression of thermogenesis in retroperitoneal white adipose tissue, which the authors show is driven by AgRP activation. Despite these changes, *AgRP-Ogt* KO mice had no food intake phenotype. However, this may be due to the genetic loss occurring early in development in this model. AgRP neuronal ablation in neonates also has little effect on feeding but when induced in adults leads to rapid starvation (112). It’s possible, therefore, that acute and post-developmental changes in AgRP OGT activity in response to ghrelin or fasting would support hunger signaling.

OGT activity may also contribute to AgRP ghrelin sensitivity through its influence on FAO capacity. In a fed or glucose abundant state, lipogenesis typically suppresses FAO through the inhibition of its gatekeeper enzyme, CPT1, by the metabolic product of the acetyl-coA carboxylase (ACC) enzyme (103). In the pre-prandial period, not only are lipogenic stimulants low but AgRP ghrelin receptor signaling potentiates FAO flux by activating AMPK to inhibit ACC through a pair of sequential phosphorylation reactions (pAMPK activated, pACC inhibited). A similar mechanism for the upregulation of FAO has been observed in peripheral tissues and is subject to regulation by *O-*GlcNAcylation. Oral *O-*GlcNAc and adipose (*aP2-cre*) GFAT overexpression (OE) increase pAMPK/pACC levels in murine liver (113) and fat (114), respectively. Importantly, these observations were made in tissues from fasted animals and in the former study, no differences were found in the livers of fed mice (113). In perfused rat hearts, GFAT inhibition (azaserine) depresses pAMPK/pACC and reverses GlcN’s potentiation of FAO (115). AMPK activity may be further driven by a potentiating *O-*GlcNAcylation of the AMPK α1 catalytic subunit, which was increased in the adipocyte GFAT OE model (114). Like the Kv7.3 results, these data suggest that *O-*GlcNAcylation in AgRP neurons may maintain their physiological functions, including orexigenesis and lipid sensing.

Nevertheless, some studies have characterized a negative relationship between OGT activity and the regulatory mechanisms of FAO. Multiple models targeting OGT or OGA have correlated increased *O-*GlcNAc tone with a systemic shift away from FAO and towards glucose oxidation (16, 116–118). A similar respiratory shift is believed to underlie the suppression of AgRP neural activity in response to exogenously induced hypothalamic hyperlipidemia [see (119)]. In addition, the OGT/AMPK relationship is complex and contextual (120). *O-*GlcNAcylation has been shown to potentiate AMPK, as referenced above, or inhibit it, dependent on the pre-existence (or not) of activating conditions such as low glucose. Furthermore, *O-*GlcNAc promoting conditions have led to increased ACC transcription (121–125), including of the specific mitochondrial ACC2 isoform associated with CPT1/FAO inhibition (121), while direct *O*-GlcNAcylation of the lipogenically predominant ACC1 inhibits its activity in CD4^+^ T-cells (126). Notably, many of these ACC upregulations were exclusive to the fed state (123, 124), independent of AMPK activity (126), and/or associated with *O-*GlcNAc potentiated lipogenic transcription factors (e.g. ChREBP (124), SREBP1 [125)]. It is reasonable to hypothesize that these relationships are less relevant in the fasting environment of elevated AgRP OGT activity, except perhaps in the case of obesity and its effects on the fasting milieu (e.g. systemically elevated glucose/insulin/lipids and ghrelin receptor desensitization). In this case, a suppressive effect of *O-*GlcNAcylation on AgRP FAO could contribute to the nutrient insensitivity and loss of fasting-stimulated hunger associated with the obese state.

AgRP/NPY neural activity and ghrelin response underlie the pre-prandial hunger drive and are important for hypothalamic lipid sensing. Fasting and ghrelin-stimulated OGT activity in these neurons is linked to increased firing rate and ghrelin sensitivity but a direct evidential connection to consumptive outcomes has not yet been made. Nevertheless, the lipogenesis-FAO counter-regulatory relationship, which is essential for diet-related AgRP functions, is also well-documented to be under context-dependent *O*-GlcNAc control in peripheral cell types. This raises intriguing questions about the orexigenic consequences of the nutrient-uncoupled obese OGT interactome.

### Satiety

Food consumption is associated with an inhibition of orexigenic signaling and increased satiety factors. Following a meal, stomach distension and gastrointestinal nutrient signaling inhibit ghrelin and stimulate the release of satiety hormones [e.g. gastric leptin (127) and intestinal CCK, PYY, GIP (128), GLP-1 (129)]. Most of these activate local vagal nerves that run to the brainstem and regulate gastric emptying, suppress hunger signaling and promote fullness. Incretins (GIP, GLP-1) and post-prandial glucose trigger the pancreatic secretion of insulin, which is a multi-potent anorectic hormone. Insulin and incretins inhibit AgRP neurons and upregulate POMC and PVN neural activity to suppress feeding and promote systemic energy storage. Adipocyte-secreted leptin has similar effects in the brain but also acts as an insulin sensitizer and suppresses circulatory uridine (98) and orexigenic endocannabinoid level in the hypothalamus (130). Leptin does not increase acutely following a meal (131) but as a response to basal adiposity/insulin levels and influences overall perception of satiety.

As in AgRP neurons, dietary hyperlipidemia leads to a complex and multiphasic response in satiety-regulating cells. High fat consumption initially triggers a short-term drop in leptin levels that contribute to transient HFD hyperphagia (131–133) and potentiates glucose-stimulated insulin secretion to shore up nutrient uptake and storage (40, 134). Established obesity, on the other hand, is characterized by constitutive basal hyperleptinemia and hyperinsulinemia but with impaired nutrient regulation of secretion. In addition, insulin and leptin resistance develops in numerous cell types, including ARC neurons [see (101)]. Receptor resistance and numerous other cellular pathologies are exacerbated by a chronic state of systemic inflammation. Similar to orexigenic factors, then, satiety signaling in obese individuals becomes less coupled to the nutrient environment while shifts in hypothalamic insulin and leptin sensitivity contribute to an overactivation of post-prandial AgRP neurons but a suppression of POMC and PVN activity that promotes increased food intake.

Conditional OGT loss studies in the brain tie protein *O-*GlcNAcylation to hypothalamic satiety processing. Adult tamoxifen induced *Ogt* KO in the mouse forebrain (αCaMKII-CreER^T2^), including the hypothalamus, leads to transient obesity due to increased meal size and duration (135, 136). Lagerlof *et al.* attributed this to a decrease in the excitability of a subpopulation of CamKIIα^+^/OGT-deficient neurons in the PVN, re-iterating the *in vivo* phenotype through stereotactically-targeted OGT deletion in these cells (135). Additionally, they show that PVN CamKIIα^+^ (but not adjacent CamKIIα^-^) neural *O-*GlcNAcylation is bidirectionally regulated by glucose (increased) and fasting (decreased). *In vivo*, *αCaMKII-CreER^T2^ Ogt* KO prevented activation of these cells following food intake. Interestingly, hypothalamic *O-*GlcNAcylation was recently suggested to function as a type of satiation memory whereby prior nutrient intake can influence subsequent consumption and energy expenditure (137). In this model, a larger meal size induces more *O-*GlcNAcylation in brain satiety centers such as the PVN. As protein *O-*GlcNAcylation can be sustained longer than its acute nutrient stimulation, this then serves as a form of long-term potentiation, lowering the excitation threshold for subsequent satiety signaling. It would be interesting to see whether *O-*GlcNAcylation could serve as a nutrient context memory in AgRP neurons as well, for example contributing to the pro-thermogenic effect of intermittent fasting (138). Regardless, this data further supports the notion that hypothalamic OGT activity is an important regulator of homeostatic eating in both the pre- and post-prandial state.

In addition to changes in PVN *O*-GlcNAcylation and excitation, *αCaMKII-CreER^T2^ Ogt* KO mice exhibit significant neuronal cell loss in multiple brain regions. Dai *et al.* reported a 60% decrease in leptin receptor (LepR) expressing hypothalamic neurons, primarily in the ARC nucleus, at a timepoint two weeks later than used in the Lagerlof study (136). A loss of hypothalamic LepR activity is the driving cause of hyperphagic obesity in the db/db mouse model and believed to contribute to both decreased satiety and increased leptin secretion in the state of obesity. Shortly following induction, *αCaMKII-CreER^T2^ Ogt* KO mice develop systemic hyperleptinemia, prior to significant weight gain, which is consistent with a loss of LepR-mediated hypothalamic feedback inhibition over peripheral leptin production. Consequently, neuronal OGT may contribute to central satiety signaling through both leptin-dependent and independent mechanisms.

In addition to its central nervous system (CNS) effects, *O-*GlcNAcylation in the periphery directly promotes the secretion of many anorectic hormones (e.g. leptin, insulin) and is required for their upregulation by high fat feeding (24, 139). Leptin gene expression and serum levels are increased in rodent models of adipocyte hyper-*O-*GlcNAcylation including GlcN infusion (140), GFAT OE [*Glut4-cre* (141), *ap2-cre* (142)] and *Glut4-cre* OGT OE (143). By contrast, adipocyte-specific OGT loss depressed leptin transcripts and circulatory protein in HFD mice while having no effect on leptin levels in standard chow fed mice (139). These expression changes likely involve *O-*GlcNAcylation of the Sp1 transcription factor (144). Deletion of the Sp1 binding site in the leptin promoter depresses both basal and GlcN-stimulated transcription *in vitro* in 3T3-L1 adipocytes (145). Based on these studies, hyperlipidemia stimulated *O-*GlcNAcylation of adipose tissue, as described in section Lipid influence on the O-GlcNAc regulatory system, is a plausible contributor to progressive hyperleptinemia in obese individuals.

In the last decade, a considerable literature has developed to support the requirement of β-cell OGT activity for the efficient and lipid-responsive secretion of insulin. β-cell *Ogt* KO mice (*Rip-cre*) are severely hypoinsulinemic due to depressed β-cell mass and insulin transcription as well as proinsulin maturation deficits (146). This has been attributed, in part, to *O-*GlcNAc regulation of the transcription factor Pdx1, a master regulator of both pancreatic and β-cell development and function [see (147–149)]. Pdx1 *O-*GlcNAcylation increases its DNA binding and potentiates insulin secretion in Min6 insulinoma cells (150). Pdx1 protein levels were also depressed in OGT depleted β-cells (146, 151). In addition, *O-*GlcNAcylation of the translation initiation protein eIF4G1 has been implicated in the proinsulin processing deficits of β-cell *Ogt* KO mice, upstream of the protein stability of prohormone convertase enzyme CPE (152). More specific to the hyperlipidemic context, induced adult deletion of β-cell OGT prevents adaptive hyperinsulinemia during early high fat feeding and abolishes LCFA potentiation of glucose stimulated insulin secretion (FASIS) (24). This latter impairment was rescued by pharmacological activation of the ER calcium importer and OGT targeted protein SERCA2. Pdx1 *O-*GlcNAcylation has also been connected to FASIS through its transcriptional upregulation of the LCFA receptor GPR40 (90). Interestingly, the timing of the rise and fall of mouse islet (~80% β-cells) *O*-GlcNAcylated proteins in response to high fat feeding (24) mirrors the transient potentiation of nutrient-stimulated insulin secretion under the same conditions (24, 134). Consequently, the decrease in islet OGT activity observed in mouse and human models of progressed obesity (see **Table 1** summary) is consistent with the high proinsulin levels and acute nutrient desensitization also seen in those models.

Central and peripheral leptin and insulin resistance are also key obesity pathologies that have been definitively linked to *O-*GlcNAcylation in the latter system. Leptin and insulin receptor signaling depend on many of the same OGT targeted proteins (e.g. IRS-1, PI3K, STAT3, PTP1B). The insulin receptor is an autophosphorylating tyrosine kinase that initiates the sequential phosphorylation and activation of IRS, PI3K and especially Akt, which has a multitude of transcription and protein regulatory roles [see (153)]. LepR activation relies on ligand-stimulated JAK2 kinase, which similarly stimulates the phosphorylation of IRS/PI3K/Akt but also STAT3 and its downstream transcriptional programs [for review (154)]. Hypothalamic loss of either insulin or leptin receptors leads to hyperphagic obesity in rodents (51, 155), as does the specific disruption of leptin stimulated STAT3 phosphorylation (156). Protein tyrosine phosphatase 1B (PTP1B) is a negative regulator of insulin and leptin receptor sensitivity in multiple tissues through the dephosphorylation of IRS1 and JAK2, respectively, and is a major contributor to obesity precipitated resistance to these hormones in the brain (157, 158). PTP1B and STAT3 have been characterized as OGT targets in the liver in association with diminished insulin and leptin signaling, respectively (27, 159). In HepG2 cells, PA increased PTP1B *O-*GlcNAcylation and potentiated its activity. By contrast, mutagenic loss of PTP1B’s OGT target sites rescued hyperlipidemia induced insulin resistance. The consequences of STAT3 *O-*GlcNAcylation are more complicated. Two activating phosphorylation sites have been characterized for STAT3, pY705 and pS727, which appear differentially regulated by STAT3 *O-*GlcNAcylation under hyperglycemic conditions (159, 160). Nevertheless, Zimmerman *et al.* showed that HepG2 GFAT inhibition (azaserine) interferes with the specific leptin activation of both residues (159). Although these mechanistic investigations were conducted in liver cells, they are suggestive that excess *O-*GlcNAcylation in the hypothalamus, as much as its absence, could contribute to a loss of sensitivity to nutrient satiety signals. To that end, GlcN infusion in rats prevents leptin’s suppression of food intake (161) and a pan-neuronal loss of OGA (*Nestin*-cre) results in hyperleptinemic obese mice (162), consistent with a phenotype of central leptin resistance. Interestingly, food intake is not altered in the *Oga* KO model, which manifests during early gestation. But like the AgRP neuronal ablation models, this may speak more to the high plasticity of food regulatory mechanisms during early development. Models of adult conditional *O*-GlcNAc modification in specific populations of hypothalamic neurons under varying conditions of lipid exposure would be helpful in clarifying the role of the *O*-GlcNAc regulatory system in mediating CNS leptin and insulin sensitivity as well as homeostatic eating.

In sum, OGT appears to be required for normal satiety signaling in the brain by supporting hypothalamic excitability in the PVN and the viability of leptin sensitive neurons in the ARC. In addition, protein *O-*GlcNAcylation in adipose and pancreatic tissue supports the basal and lipid-mediated secretion of leptin and insulin as both satiety hormones and master regulators of nutrient metabolism. On the other hand, OGT activity in liver cells has been linked to insulin and leptin resistance through STAT3 and PTP1B dependent pathways that are known to drive receptor desensitization in the hypothalamus, specifically in response to obesity. The potential for *O-*GlcNAc-driven mechanisms to contribute to physiological homeostasis as well as obesogenic pathology was also noted in the previous sub-section on AgRP-mediated hunger.

### Hedonic eating

The hypothalamus is not the only brain region that contributes to food intake. Mesolimbic dopamine circuitry controls hedonic eating, associated with food cravings and motivation towards high palatability, high calorie foods. In addition, inputs from regions related to self-regulation, emotion and memory play a role, particularly in humans (163). In lean individuals, acute exposure to high fat foods triggers HFD hyperphagia due to the aforementioned changes in homeostatic signaling as well as the FA stimulated activation of hedonic circuitry. This is followed by various repressive mechanisms that work to limit prolonged or excessive caloric intake. This positive-negative feedback loop is exemplified by oral fat detection. During a meal, FAs derived from food bind to CD36 receptors on the tongue (164, 165) to stimulate mesolimbic neural activity (166). However, lingual CD36 mRNA and protein is also rapidly depressed by lipid exposure, to two-fold within an hour of re-feeding in mice (167). On a slightly longer timescale, high fat consumption stimulates the adipocytic synthesis and secretion of lipid-derived endocannabinoids (e.g. anandamide/AEA, 2-AG) that promote both orexigenic and hedonic eating drives (168, 169). In terms of negative feedback, dietary fat absorption also potentiates the secretion of the anorectic hormones insulin, GLP-1 and oleoylethanolamide (OEA), which suppress mesolimbic activation [for review (170)]. This balance of pro- and anti-consumptive factors allows for an organism to take advantage of the possibly transient availability of high caloric value foods but also to limit against the lipotoxic consequences of prolonged hyperlipidemia.

In a common theme, chronic obesity disrupts the nutrient-sensitive balance of these counter-regulatory relationships leading to a tonic overactivation of lipid-stimulated orexigenic and reward circuitry. Endocannabinoid levels increase progressively over the course of 20 wks HFD in mice (168) and, in humans, are positively correlated to markers of obesity [reviewed in (171)]. Moreover, while lean humans show a post-prandial decrease in plasma AEA and leptin levels, this does not occur in individuals with obesity (131, 172). Similarly, basal levels of GLP-1 are elevated in obese mice (173) but nutrient stimulated secretion is impaired in both mice (173) and humans (174). By contrast, circulatory ghrelin, which is typically suppressed by eating, rises in response to palatable food consumption in obese humans (174). Despite a sensitized hedonic perception of high fat foods, oral fat sensitivity and lingual CD36 expression are depressed during chronic obesity as is their dynamic regulation by dietary lipids [(175) and for review (176)]. Furthermore, central insulin and leptin resistance disinhibits dopaminergic excitability and contributes to mesolimbic overactivation (163). Thus, obesity abrogates acute nutrient feedback to the brain and shifts the motivation for eating away from homeostatic drives and towards hedonic ones that favor overconsumption of high calorie or high fat foods.

Loss of OGT in the brain and peripheral tissues lead to specific changes in HFD hyperphagia, implicating *O-*GlcNAcylation in hedonic eating. *αCaMKII-CreER^T2^ Ogt* KO mice were hyperphagic under standard chow conditions but HFD feeding failed to further potentiate this in either of the studies described previously. In fact, absolute food intake decreased in these mice a few days after switching to HFD, in contrast to the chow-fed *Ogt* KO or WT HFD control mice in the same timeframe (136). Although the underlying mechanisms have not been explicitly elucidated, the death of hypothalamic leptin-sensing neurons could have rendered them insensitive to changes in circulatory leptin levels and/or interrupted the CNS-mediated feedback loop of leptin-stimulated leptin inhibition. Alternately, OGT loss was also observed in the nucleus of the solitary tract (NTS) (135), which is the primary source of CNS GLP-1 and a critical integration site for nutrient-sensitive peripheral signals (e.g. taste, vagal afferents) and their effects on both homeostatic and hedonic neural circuits [for review (177)]. Several studies have re-iterated the importance of OGT for neuronal viability or excitability [e.g. (178, 179)] so it would be interesting to see whether *O*-GlcNAcylation in the NTS or regions of the mesolimbic system associate with dietary fat preference.

OGT loss in adipose and macrophage cells was also associated with lipid preference but in opposite directions. Fat specific (*Adipoq-cre*) *Ogt* KO mice displayed normal standard chow eating patterns but lacked HFD hyperphagia (139). The expected pattern of overconsumption was restored in these mice through dietary supplementation with monounsaturated fatty acids (MUFA). The authors show that MUFA inhibit the degradation of adipocyte AEA by fatty acid amide hydrolase (FAAH) leading to increased circulatory AEA and food intake. By contrast, adipocyte OGT loss depressed MUFA synthesis through hypoexpression of the lipid desaturase SCD, which would otherwise couple a rise in adipocyte FFA uptake to pro-consumptive signaling through suppression of FAAH. A decrease in FAAH expression or activity occurs in multiple models of obesity and HFD eating [described in (168, 180)] which, based on the pathway above, is also a predictable outcome of increased OGT activity in hyperlipidemic adipocytes. Opposite to adipocytes, OGT loss in macrophages (*LysM-cre*) stimulates HFD food intake (25). This may be related to the pro-inflammatory profile of these cells. The *LysM-cre* driver targets macrophages in the brain (181) and in the periphery, where increased circulatory cytokines can cross the blood brain barrier (182). Neural inflammation, including in response to HFD, alters dopaminergic neurotransmission and has been suggested to trigger insulin resistance in the nucleus accumbens, associated with the overconsumption of palatable foods [for review (183)]. HFD induced inflammation has also been linked to taste bud remodeling believed to underlie the gustatory desensitization of obese individuals (184). Therefore, increased macrophage OGT activity during a transient period of hyperlipidemia is likely protective against excessive inflammation and may stimulate negative feedback on overeating. However, prolonged hyperlipidemia depresses macrophage *O*-GlcNAcylation, which in combination with hyper-*O*-GlcNAcylated adipocyte secretion of AEA, would support persistently enhanced lipid uptake.

Although OGT can be linked to mesolimbic and lingual fat responses upstream of macrophage inflammatory signaling, the mechanistic pathway implicated in the *LysM-cre Ogt* KO cells suggests additional, more speculative, hypotheses. The potentiation of pro-inflammatory signaling in these cells was specifically observed downstream of the TLR4 activating drug LPS (25), in agreement with previous findings associating a TLR4 triggered suppression of macrophage *O*-GlcNAcylation with exacerbated inflammation (185). Both TLR4 receptors and OGT are expressed in mesolimbic dopaminergic neurons where they have been implicated, individually, in the perception of hedonic food reward (186, 187). More specifically, dopaminergic TLR4 activity, which is endogenously activated by saturated LCFAs, is required for dietary sugar and lipid preference in mice (186). By contrast, OGT hyperactivity in this cell type is linked to the loss of sweet taste perception in sugar fed flies (187, 188). Consequently, the mesolimbic *O*-GlcNAc regulatory system appears well positioned to mediate lipid-responsive hedonic circuitry in the brain, whether independently or downstream of TLR4-dependent signaling. In addition, TLR4 and OGT have been found in taste-responsive mammalian and/or fly tongue cells (188, 189). Both proteins are positively associated with CD36 expression and function in a variety of cell types (28, 190, 191). This specifically includes a reduction of lingual *CD36* mRNA in lipid insensitive *TLR*4 KO mice (192). While nothing is known about lipid-driven *O*-GlcNAcylation patterns in the tongue and mesolimbic neurons or the role of OGT in mediating lipid responses in those areas, these circumstantial associations warrant further investigation.

OGT activity in adipose tissue and the brain is required for transient HFD hyperphagia in rodents by supporting FA-stimulated endocannabinoid secretion and, likely, dynamic leptin signaling. Macrophage *O-*GlcNAcylation resists dietary lipid overconsumption, possibly related to anti-inflammatory effects in hedonic CNS circuitry or the tongue epithelium. However, direct OGT activity and OGT interactions with TLR4-mediated signaling in these tissues should be considered for future experimentation. The implications of persistent *O*-GlcNAcylation in adipocytes or its eventual suppression in macrophages, however, are consistent with obesogenic endophenotypes that facilitate the transition from homeostatic to hedonically dominated eating patterns that favor lipid overconsumption.

### Intestinal absorption

Once dietary lipids have reached the small intestine, the capacity of intestinal enterocytes to absorb and store them, as well as to synthesize and release TAG-rich chylomicrons, determines practical lipid uptake. Diet-derived monoacylglycerol and FFAs primarily diffuse passively across the enterocyte membrane. Inside the cell, however, they are escorted by proteins to the ER membrane for re-esterification into TAG [for review (193)]. This neutral lipid is then packaged into ER luminal LDs, used for chylomicron biogenesis, or into cytoplasmic LDs for storage. Lipids stored from prior ingestion are believed to fuel the first wave of post-prandial chylomicron secretion as well as between meal release (194). Chylomicron synthesis is closely dependent on two ER proteins - apolipoprotein B48 (ApoB48) and microsomal triglyceride transfer protein (MTTP), which facilitate formation of the pre-chylomicron phospholipid membrane and the transfer of neutral lipids from luminal LDs into its core (195). These particles mature in the Golgi, receiving additional TAG and membrane modifications, before release into the lymphatic system where they can acquire plasma-derived modifications including apoC-ii (196), which is critical for cellular uptake at target tissues. Uptake-competent chylomicrons are then secreted into the bloodstream where they can deliver diet-derived lipids to peripheral tissues.

Obesity is associated with hypertriglyceridemia due in part to increased enterocyte lipid uptake. Diet induced obesity depresses fecal lipid content in mice as a consequence of the enhanced extraction of dietary fat (197). One reason for this is an expansion of the intestinal surface area, downstream of stem cell proliferation and factors related to increased food intake (197). In addition, individual enterocytes adapted to obesity show an enhanced capacity for lipid uptake, TAG storage and secretion. Prolonged HFD, or *in vitro* PA, induces expression of enterocytic periliplin 2 (PLIN2) (198), an LD membrane protein important for long-term cytosolic LD storage [reviewed in (194)]. Two mouse models with intestinal PLIN2 depletion showed reduced cytosolic LD expansion and suppressed dietary lipid extraction under HFD conditions (197, 198). The multi-potent FA binding and transport protein CD36 has also been implicated. CD36 deficient mice and humans produce fewer and smaller chylomicrons (199–201) but the mechanisms behind this shift are not precisely delineated [for review (202)]. CD36 appears to be dispensable for net intestinal FFA uptake (199, 203). However, LCFA binding and activation of the receptor leads to the rapid induction of ApoB48 and MTTP protein (204), as well as a slower transcriptional regulation (205), but also to pre-chylomicron assembly and transport (199, 206). In lean subjects, enterocyte CD36 activation is transient due to lipid-stimulated proteasomal degradation within ~1 hour of high fat ingestion (204). However, this downregulation is absent in obese mice, leading to prolonged secretion of large particle chylomicrons (205), which both hold more TAG and are more rapidly depleted by target tissue uptake (207, 208). In sum, obesity related changes in enterocyte number, TAG storage and chylomicron secretion proteins exacerbate systemic hyperlipidemia by enhancing the capacity for dietary lipid extraction.

Although not specific to the gut, *O-*GlcNAcylation is associated with the increased stability of both PLIN2 and CD36 *in vitro*. As mentioned in “O-GlcNAc Enzymes”, OA potentiates sOGA activity and promotes its accumulation on LD membranes where it co-localizes with PLIN2 in ad-sOGA OE HeLa cells (71). RNAi knockdown of sOGA in that model stabilized PLIN2 and PLIN3 protein level through reduced proteasomal activation. OGT activity regulates the ubiquitin-dependent degradation of multiple intracellular proteins (209), including through inhibitory *O*-GlcNAcylation of the S26 proteasome itself (210–213). In HeLa cells, sOGA inhibition increased general ubiquitinylated protein abundance while its overexpression accelerated proteasomal degradation of PLIN2/3 at the LD membrane (71). It would be interesting to see whether the lipid-stimulated proteolysis of CD36 might be similarly regulated. Nevertheless, CD36 is also a direct and transcriptional OGT target. OGA inhibition (Thiamet-G) of gastric cancer cells increased CD36 mRNA and protein levels while *Ogt* KO or siRNA diminished both basal and PA-stimulated CD36 abundance (28). The transcriptional upregulation was attributed to NF-KB *O-*GlcNAcylation, which increases its activity at the CD36 promoter (28). In addition, CD36 contains at least two *O-*GlcNAcylated residues (28, 190) that, when mutated, reduce the rate of cellular FFA uptake and PA-stimulated LD accumulation (28). With the exception of sOGA, all of these proteins have demonstrated expression in enterocytes where analogous regulatory relationships, if true, would link hyperlipidemia-driven *O*-GlcNAcylation to enhanced intestinal uptake and secretion of dietary fat as well as to fat-stimulated satiety through CD36-dependent enterocyte synthesis of OEA (214, 215).

OGT has been directly knocked out in the intestinal epithelium (*Villin-cre*), which includes enterocytes, but the outcome on lipid uptake is unclear. *Villin-cre Ogt* KO mice demonstrate intestinal hypertrophy and hyperplasia (216), similar to the effects of obesity. However, in studies from the same lab, these mice also have lower body weight and improved glucose tolerance (17), inconsistent with a higher rate of nutrient absorption. Furthermore, HFD or *in vitro* short chain fatty acids (SCFAs) consistently increased intestinal *O-*GlcNAcylation (17), which argues against a potentiating role for OGT activity in expansion of the obese gut. Instead, the predominant *Ogt* KO phenotype characterized by the authors was an increase in serum GLP-1, apparently due to hyperplastic expansion of intestinal L-cells (17). This data was supported by a knockin model of rat OGT OE with transcriptional deregulation and impaired L-cell and GLP-1 levels, reflecting obesity conditions related to L-cell dysfunction [e.g. (173)]. OGT’s suppressive effects were tied in part to FOXO1 *O*-GlcNAcylation leading to the inhibition of Ngn3-mediated gene expression (17). In the CNS and periphery, GLP-1 dampens palatable food intake and potentiates glucose-stimulated insulin secretion [for review (217)]. The systemic role of intestinal GLP-1 (vs. NTS or intra-islet GLP-1) is somewhat in question, however, due to its rapid degradation in the gut and liver [see (218)]. Moreover, fecal transplantation from *Villin-Ogt* KOs to WT mice transferred the body weight and L-cell hyperplasia phenotype, implicating causative changes in the intestinal microbiome (17). The impact of any of these changes under HFD conditions is unknown. However, both gut microbiota and intestinal GLP-1 have regulatory roles in local lipid metabolism [for review (219)]. According to these studies, increased GLP-1 signaling depresses dietary fat absorption and chylomicron TAG secretion, with similar results in germ-free or antibiotic-treated animals. Therefore, HFD stimulated *O*-GlcNAcylation of the intestinal epithelium, and its subsequent effects on microbiome activity and L-cell GLP-1 hyposecretion, could conceivably contribute to increased dietary lipid extraction.

Dietary lipid absorption increases during obesity as does the secretion of TAG-rich chylomicron particles from intestinal enterocytes. Protein *O-*GlcNAcylation has not been directly linked to enterocyte physiology but OGT and sOGA activity in other cell types regulate the stability and activity of CD36 and PLIN2, which are central to hyperlipidemia adaptations in the intestine. Furthermore, *O-*GlcNAc’s transcriptional regulation of L-cell GLP-1 secretion has implications for both local lipid extraction and meal related satiety. On the basis of these mechanisms, intestinal hyper-*O*-GlcNAcylation in response to dietary or even microbiome-derived hyperlipidemia would positively contribute to the enhanced lipid uptake.

## *O-*GlcNAcylation in lipid storage and release

Section OGT activity and dietary lipid uptake described the complex and temporally dynamic relationships between dietary fat and lipid uptake, at both the motivational and absorptive level. OGT activity supports both hunger and satiety regulating mechanisms as well as lipid-potentiated hyperphagia and its feedback inhibition through dynamic, cell type-specific responses to both low and high nutrient conditions. By contrast, persistent and excessive lipid exposure overwhelms the homeostatic forces of both the lipid response and *O*-GlcNAc regulatory systems, further driving their reciprocal dysfunction. A similar pattern also emerges for the role of *O*-GlcNAcylation in peripheral lipid uptake, storage and release.

Lipids in the bloodstream are taken up by numerous cell types, some primarily for utilization (e.g. heart, skeletal muscle) and others for the purpose of storage and regulated release (i.e. white adipose tissue, liver). The source of these lipids can be diet-derived chylomicron TAG but also adipocyte-released FFAs and hepatic lipoprotein TAG, with varying contributions to fasting and post-prandial circulation. FFAs at the cell surface, either albumin-bound or lipolytically released from TAG, move across the plasma membrane with the aid of FFA binding proteins under tissue specific nutrient and hormone regulation. White adipose tissue (WAT) is the primary site for dietary lipid uptake and long-term fat storage. Adipocytes possess a specialized regulatory system that facilitates switching between meal stimulated FFA uptake and lipogenic LD expansion or fasting dominant LD TAG lipolysis and FFA secretion. Furthermore, WAT storage capacity can expand to accommodate a higher lipid load through adipogenesis and hypertrophy. The liver is also a major source for nutrient uptake and short-term storage as well as producing energy-generating substrates when exogenous sources are low. This includes a careful regulation of interconnected lipid and glucose metabolic pathways. Due to these dual roles, WAT and the liver are particularly sensitive to intra- and extracellular cues that differentiate the post-prandial and fasting state. Under physiological conditions, *O*-GlcNAcylation facilitates adaptive nutrient uptake, production and storage but differentially between tissues according to their specific roles throughout the day or over a developmental time course. In obesity, the signals that guide dynamic *O*-GlcNAc activity, substrate targeting and functional outcome become less context-specific such that static and tone-shifted *O*-GlcNAcylation appears to exacerbate the negative consequences of persistent hyperlipidemia.

### Cellular lipid uptake

While cellular FFA uptake can occur passively, membrane-bound and adjacent proteins facilitate transport efficiency in both generic and tissue-specific ways. Circulatory TAG is first be hydrolyzed by endothelial lipoprotein lipase (LPL), which is activated by ApoC-ii proteins in the lipoprotein membrane. The LPL protein is synthesized in capillary-adjacent cells then transported to the epithelial wall, allowing differences in local secretion and activation to drive tissue-specific lipid uptake profiles independent of circulatory abundance [for review (220)]. LPL expression and activity is highest in white adipose and cardiac tissue (221–223). However feeding, glucose and insulin potentiate adipocyte LPL hydrolysis while these conditions are repressive in the heart (221, 224). LPL is also active in skeletal muscle lipid uptake (221, 225) but largely in response to fasting and exercise (226, 227) with little insulin sensitivity (228–230) and only a mild and transient response to eating (226, 231). Cell membrane proteins that facilitate FFA uptake (i.e. FATP1-6, FABPpm, CD36) also demonstrate context-dependent regulation. Insulin sensitive FATP1 and CD36 are particularly important in adipose, heart and skeletal muscle. Interestingly, myocyte CD36 acts as a direct FFA translocase while adipocyte CD36 facilitates insulin/lipolysis stimulated endocytosis (232, 233). FATP1 and LPL expression are low in the liver, although some evidence suggests hepatic LPL contributes to circulatory LPL levels (234). Hepatic CD36, on the other hand, is elevated during fasting (235), when FFA uptake by the liver is highest. These unique regulatory profiles help direct circulatory lipids towards vitally active cells (heart, exercising muscle) and energy production (hepatic gluconeogenesis, ketogenesis) under high demand or low nutrient conditions but also funnel the majority of post-prandial dietary fat towards WAT adipocytes that have a higher capacity for non-toxic lipogenic storage.

The machinery and rate of lipid uptake is altered by obesity in a similarly tissue-specific manner. LCFA uptake is elevated in adipose, cardiac and hepatic tissues from obese individuals (236–239) but lower in skeletal muscle (240, 241). This is consistent with the up-or-downregulation of CD36 and, excepting the liver, FATP1 in those cell types (238, 242–248). Obesity also upregulates LPL expression and activity in fat and liver while levels in cardiac and skeletal muscle are unchanged or diminished [for review (220, 239)]. However, the outcome of obesity on adipocyte lipid uptake is dependent on insulin signaling. Overweight insulin resistant individuals have reduced adipose LPL expression/activity and slower post-prandial TAG clearance compared to weight-matched but more insulin sensitive controls (249). This is likely exacerbated by obesity precipitated changes in β-cell insulin secretion (24, 40, 134) and intestinal ApoC-ii expression (204, 205, 250), namely a basal elevation with a loss of nutrient stimulation in both. During early hyperlipidemia, when insulin signaling is potentiated, these adaptations boost cardiac utilization and hepatic buffering while further directing excess dietary lipid towards adipocytes. In progressed obesity, however, the capacity for both adipose and muscle FFA uptake is reduced, exacerbating circulatory hyperlipidemia and pushing a steatogenic burden onto the heart and liver.

While investigation in this area is not extensive, changes in cellular lipid uptake proteins have been observed in *O-*GlcNAc modified models. *Oga* KO in mouse embryonic fibroblasts (MEFs) depresses transcription of *Apoc2*, *Slc27a1* (FATP1) and *Cd36* (116). These results implicate *O-*GlcNAcylation in the regulation of relevant genes but the lack of tissue specificity limits broader interpretation based on this model. By contrast, multiple investigations have demonstrated the effects of HBP-driven *O-*GlcNAcylation on CD36 in the rodent heart. As described in section OGT activity and dietary lipid uptake, CD36 is a direct and indirect OGT target and the dominant source of FFA influx in the heart. Cardiac perfusion with GlcN (190) or Gln (115) increases membrane-specific expression of cardiomyocyte CD36. Similarly, *in vitro* GlcNAc rescued a depression in CD36 *O-*GlcNAcylation and membrane expression in a model of cardiomyocyte stress (191). This data is in addition to the study referenced earlier showing that a reduction in CD36 *O*-GlcNAcylation suppresses FFA uptake rate in gastric cancer cells (28). Besides CD36, LPL has been implicated in a small number of studies. *Ogt* KO β-cells (*RIP-cre*) have fewer *Lpl* transcripts (24) while OGA inhibition increases LPL secretion in primary rat adipocytes (251). LPL in the liver has not been investigated under *O*-GlcNAc modified conditions but the *O*-GlcNAc potentiated transcription factor LXR (252), is a major driver of hepatic *Lpl* expression (253), including in response to dietary hyperlipidemia (254). Oral *O*-GlcNAc also increases hepatic FFA content, predominantly in fed mice (113). These results are largely consistent with the conclusion that OGT activity supports FFA uptake through the upregulation of LPL and CD36, notably in tissues with a hyper-*O*-GlcNAcylation response to hyperlipidemia.

It is not clear to what extent *O*-GlcNAc facilitated FFA uptake might underlie constructive hyperlipidemia adaptations, such as enhanced cardiac utilization or protective lipid sequestration by adipose and liver, as opposed to further driving lipotoxic overwhelm. However, in addition to cellular uptake proteins, HBP and/or OGT activity has been extensively linked to insulin resistance, particularly in adipose and skeletal muscle (16, 53, 142, 143, 255). OGT depletion rescues HFD insulin sensitivity in these same tissues (139, 256, 257). Therefore, elevated *O*-GlcNAc levels are expected to diminish the post-prandial and insulin-dependent stimulation of CD36 membrane expression in muscle and both CD36 internalization and LPL activation in adipose tissue in contribution to the reduced TAG clearance rate of the obese state.

The available literature supports the hypothesis that *O-*GlcNAcylation can regulate tissue specific cellular lipid uptake through the expression or activity of proteins required for lipoprotein TAG hydrolysis (i.e. LPL) and efficient FFA membrane transport (i.e. FATP1, CD36). OGT activity in adipose tissue, the liver and heart upregulate these mechanisms. Hyperlipidemia potentiates *O-*GlcNAcylation in these tissues, which may accelerate lipid clearance from the blood as an adaptive strategy to fat overconsumption or desensitize insulin stimulated uptake while potentiating basal and ectopic uptake, further driving serum hyperlipidemia and pathological lipid accumulation.

### Adipocyte lipolysis

Adipose tissue is a dominant contributor to post-prandial lipid uptake and fasting FFA secretion, making its capacity for lipid storage and metabolic flexibility critical for systemic nutrient homeostasis. As in most cell types, WAT FAs are packaged into cytosolic LD TAG, which under physiological conditions constitute ~ 80% of adipocyte weight (258). TAG content is dynamically controlled by LD membrane proteins including lipogenic acyltransferases, lipolytic lipases and PLINs. There are multiple PLIN isoforms associated with WAT LDs that take on structural, signaling and regulatory roles [see (259)]. PLIN1, which is predominantly expressed in adipocytes, is important for the regulation of lipolysis. TAG lipolysis occurs through the sequential action of three lipases (ATGL, HSL, MGL), which progressively liberate one FFA from each TAG fatty acyl chain [for review (260)]. In the basal lipolytic state, low ATGL activity on the LD membrane provides constitutive FFA flux. Fasting and exercise enhance the lipolytic rate, however, by triggering sympathetic catecholamine release to stimulate adipocyte β-adrenergic receptors, leading to the cAMP/PKA-mediated phosphorylation of PLIN1 and HSL. Phosphorylated PLIN1 dissociates from the ATGL co-activator protein CGI-58, freeing it to potentiate ATGL’s catalytic activity up to 20-fold (261). Between rising levels of diacylglycerol and its own phosphorylation, cytoplasmic HSL translocates to the LD surface to further promote lipolysis [for recent review (262)]. By contrast, post-prandial insulin signaling suppresses lipolysis by triggering HSL dephosphorylation and cAMP degradation and by interfering with the PKA phosphorylation of PLIN1 (263) as well as by blocking CD36 dependent FFA exocytosis (233). Moreover, insulin drives LD expansion by amplifying FFA uptake, acyltransferase activity, lipogenic gene expression and glucose-derived lipogenic substrate availability [for review (264)]. These regulatory relationships ensure that the balance between adipocyte FFA storage and release is matched to the acute nutrient conditions of the body.

Obesity is associated with an increase in basal lipolysis and a decrease in catecholamine-stimulated FFA secretion (260). Increased leptin levels, described in the previous section, and a reduced LD membrane density of PLIN1 contribute to higher constitutive FFA release (265, 266). The hypoexpression and protein degradation of PLIN1 may be downstream of pro-inflammatory cytokine TNFα (266, 267), which has a higher adipose tissue concentration in obese individuals (268). At the same time, changes in adrenergic receptor expression/composition, associated with a reduction in cAMP generation, as well as decreased HSL transcription have been blamed for the blunting of stimulated lipolysis (260). Insulin receptor resistance, also associated with inflammatory cytokines in obesity (269), is expected to potentiate both these lipolytic dysregulations, exacerbating both adipose and hepatic lipid accumulation while diminishing the validity of serum FFA level as a marker of the fasting state.

The adipocyte *O-*GlcNAc regulatory system influences LD FFA storage and release through effects on PLIN1. Yang *et al.* have shown that OGT and PKA compete for two of the same PLIN1 target sites, S517 and S492, with opposing effects on the stimulated activation of lipolysis (16). Accordingly, adipocyte OGT loss (*Adipoq-CreER*) increases the interaction between CGI-58 and ATGL on the LD surface and enhances both fasting and β-adrenergic stimulated lipolysis. Consistently, OGA inhibition protects WT adipocytes from forskolin (i.e. cAMP) induced LD depletion. Furthermore, overexpression of rat OGT in mouse adipose tissue exaggerates HFD WAT expansion and diminishes *in vitro* stimulated lipolysis but only in adipocytes derived from HFD fed animals. Surprisingly, *O-*GlcNAc modifying conditions have similar effects on the LD dynamics of HeLa cells, which do not express PLIN1. Oleic acid loading followed by serum depletion (i.e. cellular fasting) triggers lipolytic LD depletion in WT but not OGT OE HeLa cells while LD loss is exaggerated in OGA OE cells (16). This could be attributable to sOGA mediated proteolytic degradation of PLIN2 and PLIN3 (see “Intestinal Absorption”). Like HeLa cells, sOGA in 3T3-L1 adipocytes co-localizes with PLIN2/3 but only on smaller, nascent LDs while sOGA is absent on larger PLIN1-coated LDs (71). The switch from statically lipogenic PLIN2 to lipolytically dynamic PLIN1 is a physiologically important component of LD maturation [for review (270)]. Some evidence suggests that PLIN2 and PLIN1 compete for occupancy in the LD membrane (271), in which case adipocyte sOGA might facilitate PLIN1 expression and LD maturation by stimulating PLIN2 degradation. To that point, OGA inhibition (NButGT) during 3T3L1 differentiation decreases PLIN1 levels and LD biogenesis *in vitro* (272). Physiologically, PLIN1 also plays a role in suppressing constitutive lipolysis but the consequences of OGT/OGA activity on this have not been directly studied. Basal lipolysis is not significantly altered in the models described above but was found to be elevated by GFAT inhibition in cultured human adipocytes (273). Nevertheless, sOGA and OGT activity in adipocytes appears to support adaptive functioning by adjusting lipolytic sensitivity while hyper-*O-*GlcNAcylation in obesity may limit metabolic flexibility by suppressing LD maturation and PLIN1-mediated lipolysis.

In addition to direct effects, OGT-driven changes in adipose insulin sensitivity and macrophage cytokine secretion may further fuel lipolytic dysfunction in obesity. Adipocyte insulin resistance in models of elevated HBP flux (142, 255, 274, 275) was among the first pieces of evidence linking *O-*GlcNAcylation to insulin sensitivity. *In vivo*, this may be restricted to the context of hyperlipidemia as numerous adipocyte OGT models fail to impact insulin sensitivity in standard chow fed mice (16, 139, 276). By contrast, the same studies show that under HFD conditions, adipose OGT OE (rat OGT knockin) worsens insulin resistance (16) and *Ogt* KO (*adiponectin-cre*) prevents it altogether (139). These models are also associated with changes in lipolysis or circulating FFA levels consistent with an exacerbating role for OGT in obesity related lipolytic dysfunction. Another pathway towards this conclusion is through *O-*GlcNAc regulation of macrophage cytokine transcription. As previously described, prolonged HFD depresses macrophage *O-*GlcNAcylation, correlated to pro*-*inflammatory polarization and cytokine secretion in adipose tissue (25). Obesity substantially increases adipose macrophage content, from ~10% up to 40% (277), accelerating the local (and systemic) inflammation that also contributes to insulin desensitization (269). Consistent with cytokine induced insulin resistance, WAT from *LysM-cre* macrophage *Ogt* KO mice show increased β-adrenergic stimulated lipolysis (25). Similarly, SV-differentiated WT adipocytes co-cultured with *Ogt* KO macrophages exhibit stronger lipolytic activation to acute inflammatory stimulation (25). Thus, adipocyte and macrophage *O*-GlcNAc responses to prolonged hyperlipidemia, despite their opposite directions, likely both contribute to adipose tissue insulin resistance and the dysregulation of lipolysis in obese individuals.

### Adipogenesis

Adipose tissue adapts to persistent nutrient excess by first expanding individual cell size (hypertrophy) and subsequently the number of mature adipocytes (hyperplasia) [for review (278)]. Moderate hypertrophy, which is driven by LD expansion, preserves systemic insulin sensitivity and protects more vulnerable tissues against lipotoxicity. However, very large adipocytes develop significant stress (mechanical, hypoxic, ER, oxidative, etc.) that leads to intracellular insulin resistance and necrotic features [reviewed in (279)]. This reduces adipose tissue lipogenic and lipolytic capacity and stimulates inflammatory cytokine secretion. As a mitigating strategy against excessive hypertrophy, hyperplasia generates new adipocytes from a pool of precursor stem cells.

Adipogenesis begins with the irreversible commitment of stem cells to a pre-adipocyte form (279). When triggered to differentiate, pre-adipocytes undergo clonal mitotic expansion followed by terminal differentiation, the latter characterized by growth arrest, morphological changes, LD formation/maturation and adipokine synthesis. Terminal differentiation begins with the transient expression and activation of the beta and delta isoforms of CAAT/enhancer-binding proteins (C/EBP), which initiate expression of *Pparɣ* and *C/ebpα* during a first transcriptional wave (278). During the second wave, C/ebpα and PPARɣ (+co-activator RXR) act in a positive feedback loop to rapidly promote their mutual expression. Subsequently, they work cooperatively to promote the gene expression of lipogenic and adipokinic proteins. Adipogenic success, therefore, influences both the size of the adipocyte pool and the capacity of newly differentiated adipocytes to take up, store and release FFAs through their expression of relevant enzymes and regulatory proteins. By contrast, decreased adipogenesis and increased hypertrophy contribute significantly to metabolic dysfunction in progressed obesity.

Dynamic *O-*GlcNAcylation is a hallmark and requisite for adipocyte differentiation. Ishihara *et al.* were the first to demonstrate that *O-*GlcNAcylation increases progressively over the course of an 8-day 3T3-L1 differentiation. In that study, OGT protein expression mirrored *O-*GlcNAcylation while OGA was upregulated on day 1 then remained at that level throughout (280). This *O-*GlcNAcylation pattern was recapitulated by Hsieh *et al.*, who also showed that *Gfat* mRNA and protein were increased but in a cyclical pattern, with transient protein peaks on days 1 and 4 (281). Multiple studies have noted that different target proteins appear to be *O-*GlcNAcylated at different timepoints (71, 273, 280, 281), with several heavier weight molecular bands only appearing in western blots of differentiated cells. Among the identified OGT targets in these studies were proteins involved in nutrient metabolism, cytoskeletal structure, nuclear transport and transcription regulation (280), including PPARɣ and C/EBPβ (281). Inhibition of GFAT (DON, azaserine, siGFAT) prevented the differentiation-induced increase in *O-*GlcNAcylation and forestalled adipogenesis based on the absence of phenotypic markers (morphology, LD accumulation), transcriptional markers (*C/ebpβ*, *Pparɣ*, *Srebf1*) and functional markers (*Ir*, *Glut4*, *Fas*, *Adiponectin*, *Plin1*, *Plin2*) (280, 281). Nevertheless, OGA inhibition has also been linked to depressed differentiation, although not consistently across studies. Applied during 3T3-L1 differentiation, pharmacological OGA inhibition by NAG-thiazoline delayed *Pparɣ* and *ap2* expression in one study (282) but NButGT did not in another (272). However, the latter investigation did note that 3T3-L1 lipid accumulation and PPARɣ transcriptional activity were decreased. On the other hand, gene trap *Oga* KO WAT cells, generated from differentiated MEFs, showed no differences compared to WT controls (118). Given the importance of transient, timed protein activity for differentiation to progress, it is not surprising that OGT targeting shifts over time and that disruption of that timing, by elevating or depressing global *O-*GlcNAcylation, would impair adipogenesis.

PPARɣ and C/EBPβ are strongly *O-*GlcNAcylated during normal 3T3-L1differentiation. Activation of either of these transcription factors is sufficient to independently trigger adipocyte differentiation but only PPARɣ is essential for it to occur ( (283) ISBN 9780128146491). PPARɣ is expressed in two splice isoforms. The adipocyte-exclusive PPARɣ2, which is *O-*GlcNAcylated at Thr84, is the isoform required for adipogenesis (139, 284). Mutagenic loss of T84 *O-*GlcNAcylation decreases PPARɣ2 protein and activity by 45% (ligand-stimulated) to 74% (basal) in transfected HEK293 cells (139). Similarly, HFD adipose tissue from *Adipoq-cre Ogt* KO mice show depressed PPARɣ2 levels with increased inhibitory phosphorylation at Ser82. The PPARɣ1 isoform is more broadly expressed across tissues, including in adipocytes, but its functional role is not well understood (284). Thr54 is an OGT target in PPARɣ1 but increasing its *O-*GlcNAcylation (NButGT) reduces enzymatic activity (272), making its contribution to adipogenesis unclear. It is worth noting that elevated glucose during 3T3-L1 differentiation increased the *O-*GlcNAcylation of PPARɣ1 but not PPARɣ2 in the same study, suggesting independent regulation of the two isoforms.

**Figure 3:**
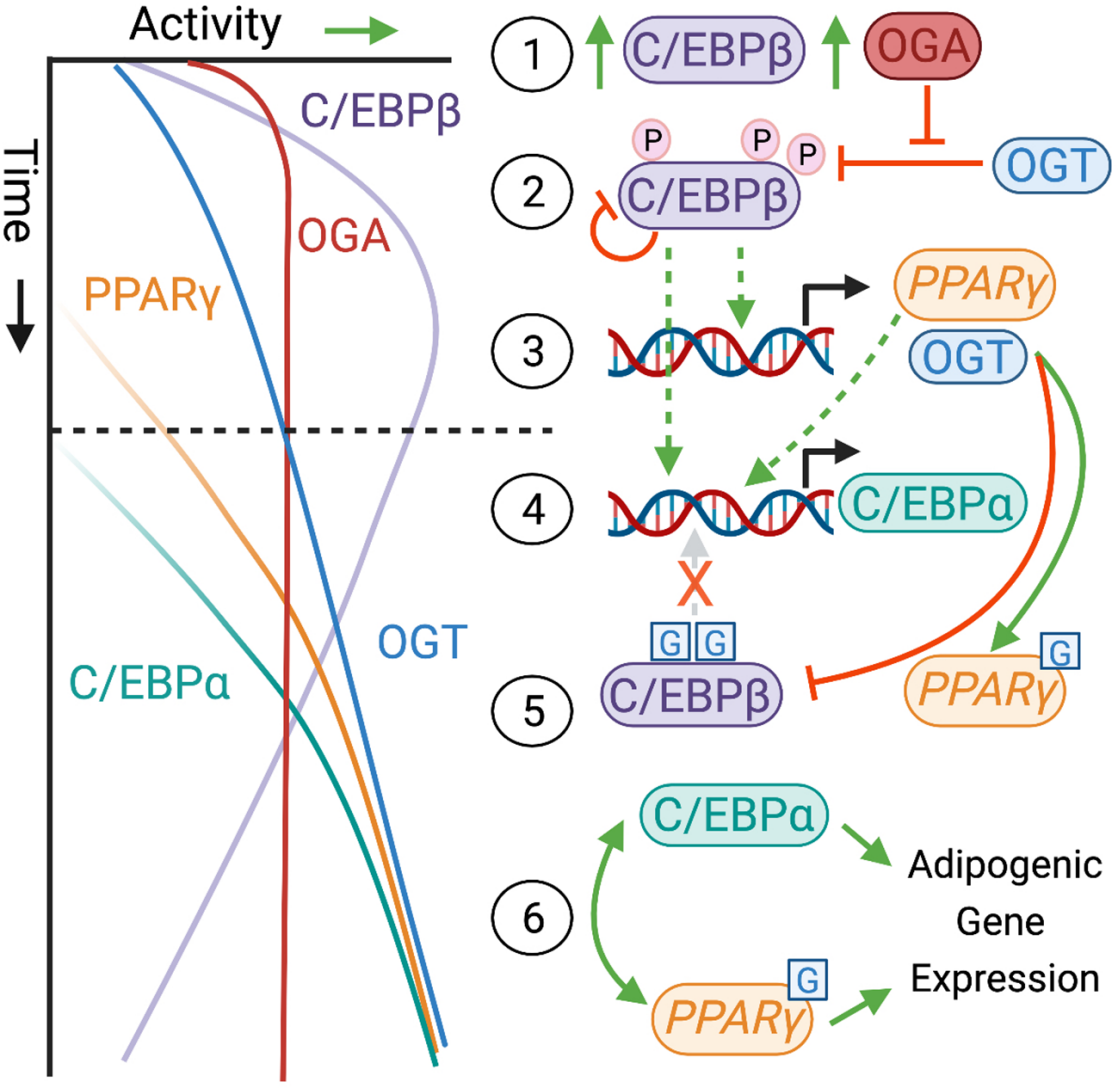
Model for *O-*GlcNAc Regulation of Adipogenesis. Adipocyte terminal differentiation is characterized by a progressive increase in *O-*GlcNAcylation but the precise rationale for this is not fully elucidated. One possibility is that a high OGA/OGT ratio at the start of differentiation (1) permits C/EBPβ kinase activation by keeping *O-*GlcNAcylation off of nearby inhibitory residues (2). Activated C/EBPβ can then act at the *Pparɣ* and *Ogt* promoters (3). Both C/EBPβ and PPARɣ stimulate expression of C/EBPα (4), which marks the transition from the 1^st^ to the 2^nd^ transcriptional wave (—–) on ~ day 3 of a standard 8-day 3T3L1 differentiation protocol [see (272, 280, 282)]. Building OGT levels, with static OGA expression, could then provide negative feedback on C/EBPβ activity and simultaneously potentiate PPARɣ through their respective *O-*GlcNAcylations (5). In the final phase of differentiation, C/EBPα and PPARɣ act as transcription factors for each other and for adipogenic and adipokinic genes that characterize a mature adipocyte (6). The left side graph shows the approximate relative activity of each protein (x-axis) over an 8-day time course (y-axis). On the right, solid arrows represent positive (green, –>) and negative (red, –|) effects. Dashed arrows indicate translocation. Phosphorylation is shown as an encircled pink P and *O-*GlcNAcylation as a blue square with a G. Created with BioRender.com

C/EBPβ is *O-*GlcNAcylated at two adjacent serine residues (Ser180, Ser181) (282). When occupied, these sites prevent the sequential activating phosphorylations of C/EBPβ, first by MAPK/CDK2 (Thr188) and then by GSK3β (Ser184, Thr179). These modifications are required for C/EBPβ to bind to DNA during the early stage of terminal differentiation. Consequently, OGA inhibition delays the expression of C/EBPβ differentiation targets, *Pparɣ* and *ap2*, while mutagenesis of the S180/181 residues strongly potentiate C/EBPβ transactivation of the *C/ebpα* promoter. Therefore, it is likely that rising *O-*GlcNAcylation plays a constructive role in terminating the transient elevation of C/EBPβ activity during the first transcriptional wave and enhancing PPARɣ2 function in the second wave of adipocyte differentiation. Interestingly, the *Ogt* promoter contains a binding site for C/EBPβ which is required for its transcriptional potentiation by OGA and p300 (an acetyltransferase C/EBPβ co-activator) in transfected HEK293T and pancreatic cancer cells (285). OGA, p300 and C/EBPβ are all elevated during early adipocyte differentiation (280, 286) whereupon they could mediate the transition from the first to the second transcriptional wave by facilitating early C/EBPβ driven transcription, including *Ogt* expression, that then provides negative feedback on further C/EBPβ activity. A simplified model of this regulatory hypothesis is summarized in **Figure 3**. Unfortunately, the effect of obesity on *O-*GlcNAcylation patterns in differentiating adipocytes is not known but the oppositional effects of *O-*GlcNAcylation on C/EBPβ and PPARɣ in relation to the timing of their adipogenic activities suggests that any shift in the ratio of OGT/OGA activity could delay or inhibit hyperplastic expansion.

Dynamic activity of the *O-*GlcNAc regulatory system in adipose tissue contributes to constructive adaptations to transient hyperlipidemia. These include modulating the activation threshold for PLIN1 mediated lipolysis (see previous sub-section) and controlling the activity of adipogenic transcription factors that permit expansion of WAT storage through hypertrophy and hyperplasia. At the same time, persistent elevations in adipocyte *O-*GlcNAc tone as well as macrophage OGT depletion in the face of prolonged overnutrition/obesity are implicated in adipose tissue dysfunctions including a loss of nutrient sensitive lipolysis, increased inflammation, excessive lipid retention, impaired adipocyte differentiation and both local and systemic insulin resistance.

### Liver

The liver is an important tissue for overflow nutrient uptake and in the maintenance of fasting nutrient availability. Hepatocytes take up TAG-depleted chylomicron remnants and FFAs from the circulation but also generate *de novo* FFAs when glucose is abundant. FFAs that are not oxidized in the liver are sequestered into TAG and either stored in cytoplasmic LDs or packaged for secretion into very low density lipoproteins (VLDL). The capacity for hepatic TAG storage under physiological conditions is limited and the majority is released back into the bloodstream in response to elevated FFA uptake (287, 288) or VLDL-TAG accumulation [for review (289, 290)]. Consequently, VLDL synthesis, which largely mirrors chylomicron biogenesis and its dependency on MTTP and ApoB availability (see “Intestinal Absorption”), is closely tied to hepatic TAG release. Unlike in enterocytes, however, insulin limits hepatic TAG secretion by suppressing ApoB100 translation and stability [reviewed in (291)] and by excluding MTTP transcription factor FOXO1 from the nucleus (290). Even so, hepatic cytosolic TAG storage is highest during physiological fasting [e.g. (124)] and VLDL secretion contributes significantly to circulatory TAG in the late post-prandial period (292). It should be noted that the liver plays a central role in the synthesis, storage and post-absorptive release of glucose as well, which is heavily interconnected with lipid metabolism [recently reviewed in (293)]. Like adipose, then, the liver depends on insulin and nutrient sensitivity, lipogenic capacity and metabolic flexibility to maintain control over systemic nutrient homeostasis.

Both hepatic fat content and circulatory VLDL-TAG increase in response to obesity. Lipid uptake to the liver is potentiated, as previously discussed, but *de novo* lipogenesis also increases. This is partly because insulin’s potentiation of lipogenic gene expression is selectively preserved (294) even as VLDL secretion and then gluconeogenesis become disinhibited (290). Synthesized FFAs typically contribute to <5% of circulatory TAG in metabolically healthy fasted individuals (295). However, that proportion increases under hyperlipidemic insulin resistant conditions (295) and in response to dietary lipogenic stimulation (296). Moreover, TAG incorporation of saturated long-chain FAs, which are abundant in high fat foods, favors higher VLDL release [reviewed in (297)]. Dietary hyperlipidemia, then, initially elevates both fasting and post-prandial TAG secretion, sparing the liver from excessive accumulation. Prolonged exposure of hepatocytes to high FFA concentrations, however, triggers ER stress and ApoB100 degradation leading to enhanced lipid retention (287). Furthermore, disturbances in glucose and lipid metabolism positively feedback on each other, with hyperglycemia driving lipogenesis and lipid accumulation disrupting both hepatic and whole-body glucose metabolism [for review (293)]. The liver thus becomes both a victim of and a source for pathological hyperlipidemia and systemic metabolic dysregulation in progressed obesity.

Lipid handling by the liver incorporates many elements of FFA uptake, hormone receptor sensitivity and lipoprotein synthesis already discussed, including their susceptibility to regulation by *O-*GlcNAcylation. sOGA stimulates the proteolysis of PLIN2, which is highly abundant on liver cell LD membranes (298, 299). PLIN2 OE *in vitro* suppresses VLDL secretion by recruiting FFAs into cytosolic storage (300), a pathway implicated in hepatic lipid accumulation following diet-induced obesity (301). *O*-GlcNAc stabilization of PLIN2, though not yet demonstrated in the liver, could contribute to cytosolic lipid hoarding, as could an OGT-driven increase in CD36 activity and expression. The facilitative role of OGT in PTP1B-driven insulin resistance has been directly demonstrated in the liver. As described in “Satiety”, PTP1B is a multi-tissue negative regulator of insulin and leptin signaling and its expression is positively correlated to VLDL secretion in the rodent liver (302). Reduced *O*-GlcNAcylation of PTP1B improves HepG2 insulin sensitivity and decreases lipid accumulation following PA enrichment of the media (27). However, PTP1B *O-*GlcNAcylation is specifically increased in the liver of HFD mice (27). Hepatic insulin resistance in the context of obesity increases the nuclear localization of FOXO1 and the expression of its gluconeogenic and lipogenic gene targets (303), driving up VLDL-TAG secretion (304). High glucose also potentiates hepatic FOXO1 transcription through *O*-GlcNAcylation of nuclear-permitted protein (305, 306). These conditions are potentially additive in progressed obesity, although the effects of OGT activity on non-gluconeogenic FOXO1 gene expression (e.g. MTTP) have not been tested. Consistent with these mechanisms in principle, however, both liver Ad-OGT OE (89, 124) and human *PEPCK-cre* GFAT OE (307) mice are insulin resistant with disturbed hepatic and systemic glucose metabolism as well as fasting or random-fed plasma hypertriglyceridemia. By contrast, Ad-OGA OE, targeted to the liver, rescues plasma/hepatic TAG levels and glucose tolerance in db/db obese mice (124), implicating static hyper-*O*-GlcNAcylation of the liver as a contributing factor to local and circulatory hyperlipidemia in obesity.

In addition to modulating the response to extrahepatic nutrients and hormones, OGT activity drives *de novo* lipogenesis in the liver through posttranslational enzyme targeting. Among the most important lipogenic enzymes are FAS and ACC, whose regulation by OGT was referenced in earlier sections. FAS has an intriguingly reciprocal relationship to OGT and OGA, as it binds both enzymes to influence their activity as well as its own (see “*O-*GlcNAc Enzymes”). Hepatic FAS is directly *O-*GlcNAcylated, more so in re-fed vs. fasted liver or in fasted livers from obese vs. lean mice (87). OGA inhibition upregulates FAS protein levels in human hepatocyte cells by increasing its interaction with the deubiquitinase USP2A (87). Moreover, an increase in FAS-dependent PA accumulation in non-alcoholic fatty liver disease (NAFLD) liver cancer cells was attributed to higher OGT levels (308). Directly upstream of FAS in the lipogenesis pathway is ACC, whose AMPK-dependent inhibition by OGT was discussed in “Hunger”. However, these studies were reported in the context of FAO activation, where the mitochondria bound ACC2 paralog (309) is the more likely target. By contrast, cytoplasmic ACC1 (309), contains multiple OGT target sites that potentiate its activity, independent of AMPK, in CD4^+^ T cells (126). As an alternate route to the same end, *O*-GlcNAcylation of inactivated AMPK is inhibitory *in vitro* and associated with ACC disinhibition (79). This mechanism was blamed for the correlation between OGA inhibition and reduced pAMPK/pACC in fish liver (31). These post-translational relationships provide a mechanism for rapidly amplifying lipogenesis in response to the specific combination of elevated nutrient levels and OGT activity in the liver.

Nutrient-sensitive gene expression is another major regulatory pathway for hepatic lipogenesis [for review (290)]. Nearly all of the principal transcription factors for this are *O-*GlcNAc regulated including ChREBP, LXRα, SREBP-1c, and FXR. Liver ad-OGA OE in db/db obese mice diminishes total and *O-*GlcNAcylated ChREBP levels with a concomitant decrease in transcriptional targets, FAS and ACC (124). Ad-OGT OE in WT mice has the opposite effect, potentiating ChREBP *O*-GlcNAcylation and lipogenic enzyme transcription (i.e. *Fas*/*Acc*/*Scd1*), as well as hepatic lipid accumulation (124). However this phenotype was only observed in tissues from fed mice, emphasizing the importance of insulin and nutrient availability in mediating OGT’s pro-lipogenic effects. Interestingly, the feeding-stimulated *O*-GlcNAcylation of nuclear ChREBP in the liver is dependent on LXR (93), either due to its recruitment of OGT to the nucleus (see “*O*-GlcNAc Enzymes”) or its role as an upstream transcription factor. LXR is itself *O*-GlcNAcylated in response to elevated glucose (93, 252), which also potentiates its transactivation of SREBP-1c (252). Although not directly *O*-GlcNAcylated (93, 124, 125), hepatic SREBP-1c is further supported by OGT-dependent protection against proteasomal degradation (125). The nuclear receptor FXR regulates bile acid metabolism but also targets proteins involved in lipogenesis and VLDL synthesis and acts as a transrepressor of ChREBP at some promoters [for review (310, 311)]. FXR *O-*GlcNAcylation and transcriptional activity is increased in glucose-treated HepG2 cells or re-fed mouse liver (312). All of these glucose and/or insulin stimulated proteins contribute to normal glucose-derived lipid storage in the liver, which is likely facilitated by post-prandial OGT activity. In obesity, however, persistent *O*-GlcNAcylation may exacerbate both hyperlipidemia by facilitating hepatic insulin resistance to VLDL repression and amplifying *de novo* FFA and VLDL production.

While post-prandial conditions potentiate the *O*-GlcNAcylation of most of the hepatic proteins described above, prolonged fasting is the driver in other cases. Both p53 and PGC1-α are more *O*-GlcNAcylated in fasted (24 hrs) vs. re-fed liver (11, 313). These proteins share a common regulatory target with fasting glycosylated AgRP neurons (see “Hunger”). Namely, their enhanced activities in this context all potentiate gluconeogenic gene expression, dependent on their association with OGT (11, 313). In the p53 and AgRP studies, starvation is associated with an increase in OGT protein levels (111, 313). This has also been seen in glucose deprived cells *in vitro* (37, 314, 315) and variously attributed to AMPK-dependent (37) or GlcNAc-mediated (314) *Ogt* transcription. Ruan *et al.*, however, showed that HCF-1 helps recruit OGT specifically to PGC1-α, with peak *in vitro* effects near average basal blood glucose levels (i.e. ~5 mM) (11). This is consistent with the expected range for physiological gluconeogenesis regulation. Shifts in hepatic OGT targeting have also been seen in response to fasting elevations of glucagon (316). In that study, glucagon receptor activation led to CaMKII phosphorylation of OGT and its increased pro-activating association with autophagic protein ULK1 (316). Moreover, starvation (24-48 hrs) of liver-specific ad-*Ogt* KO mice resulted in fewer autophagy markers and reduced hepatic glucose and FFA content (316), the latter presumably due to reduced LD catabolism. Hepatic lipophagy has been observed as early as 6 hrs after fasting, believed to contribute additional substrate for FAO (317). p53 activity also upregulates multiple proteins that promote FAO and negatively regulate FAS and SREBP-1c [for review (318)]. Ad-PGC1-α OE in the rat liver potentiates FAO and diminishes hepatic and plasma TAG levels (319). Although gluconeogenesis has been the primary endpoint assessed in models of low nutrient hyper-*O*-GlcNAcylation, it would be interesting to see whether the fasting-appropriate shifts in lipid metabolism associated with the activation of these proteins is similarly upregulated.

Like the ARC hypothalamus and adipose tissue, the liver has dual roles, both reigning in post-prandial nutrient excess and ensuring a systemic supply of energy substrates (i.e. glucose, FFAs, ketones) during the post-absorptive and fasting periods. Feeding-stimulated *O*-GlcNAcylation of glucose and insulin sensitive targets facilitates nutrient storage by potentiating lipogenic transcription factors and enzymes. Fasting also induces *O*-GlcNAcylation of select targets involved in glucose and energy production, suggesting the importance of dynamic OGT activity for metabolic flexibility in this tissue. Consequently, static hyper-*O*-GlcNAcylation in obesity is likely to contribute to hepatic steatosis, circulatory hyperlipidemia and hyperglycemia by over-driving lipogenesis and glucose production while accelerating insulin resistance that disinhibits VLDL-TAG secretion.

### Postprandial vs. fasting state

An important theme throughout this review is that the regulation and consequences of OGT activity differ in fasting and fed tissues as well as between physiological and obese conditions. For example, OGT OE in the mouse liver only manifests as hepatic steatosis in the fed state (124) while the lipolytic phenotypes of OGT KO in adipose (16) or kidney proximal tube epithelial cells (15) are stronger in fasted mice. Moreover, OGT loss facilitates fasting lipolysis in the first tissue but inhibits it in the second. This type of tissue specific outcome may help explain why whole body null mutations of either *ogt* or *oga* produce similar lipid storage deficiencies in *C. elegans* (320, 321). Cell type discrepancies are also apparent in the *O*-GlcNAc regulatory system’s response to systemic nutrient status. Fasting decreases the overall *O*-GlcNAcylation of inguinal WAT in mice without effecting epididymal WAT (16). In channel catfish, fasting induces *Ogt* mRNA in the liver, with no significant change in muscle or brain, while overfeeding selectively potentiates *ogt* expression in the brain (322). And as discussed in the previous section, even the OGT targeting of different proteins within the same cell can be reciprocally regulated by fasting and feeding to drive diverse metabolic adaptation strategies. These findings illustrate the complexity of the *O*-GlcNAc regulatory system as a dynamic mediator of nutrient context homeostasis that depends on unique interactions with extracellular signaling and the intracellular environment to achieve the right outcome at the right time for different tissues. By contrast, the metabolic dysfunctions that arise from persistent hyperlipidemia dull the distinction between fasting and post-prandial conditions, setting the stage for *O*-GlcNAc confusion to exacerbate obesity pathology.

The post-prandial state is marked by high levels of circulatory glucose and insulin as well as increased ER stress that coincide with an overall elevation in meal-related glycosylation. Following nutrient consumption, elevations in *O*-GlcNAcylation are driven by the mass action of enhanced glycolytic flux, which potentiates not just HBP activity but all glucose derived metabolic branches. Among these, NADPH (from the pentose phosphate pathway) and acetyl-coA (from glucose oxidation) act as substrates for FAS in the initial steps of *de novo* lipogenesis. FAS activation stimulates OGT protein expression and sets up a positive feedback loop between the two, enhancing lipogenic flux and suppressing FAO and changing the relative abundance of lipid metabolites. Evidence for the direct and indirect effects of different lipid species (e.g. palmitate, LCFA-CoAs) on the *O*-GlcNAc regulatory system and its modifiers was reviewed in section Lipid influence on the O-GlcNAc regulatory system. Insulin signaling alters the OGT substrate landscape through numerous effects on transcription (e.g. ChREBP), protein stability (e.g. CD36) and localization (e.g. FOXO1). However, it also potentiating OGT activity through tyrosine phosphorylation (323) and directs its translocation to cell membranes, particularly lipid rafts (92) that play a significant role in protein complex assembly and signal transduction [for review (324)]. In a detailed characterization of feeding-related *O*-GlcNAc regulatory patterns in the fly body, Liu *et al.* demonstrate how both eating and circadian rhythmicity drive transient elevations in pre-prandial Ogt and fasting Oga (64). These cycles guide global *O*-GlcNAcylation levels to rise and fall *in tandem* with eating (64), evidencing the important role of this modification in the ingestion and post-prandial response to food.

Moreover, insulin signaling has a particular impact on nutrient-GFAT coupling. As mentioned in section Lipid influence on the O-GlcNAc regulatory system, hyperphysiological levels of insulin appeared to be required for lipid infusion to potentiate HBP product synthesis in skeletal muscle (52, 53) and obesity was associated with higher skeletal muscle UDP-GlcNAc in two hyperinsulinemic mouse models (49, 50) but not in insulin deficient Zucker Diabetic Fatty rats (54). In humans, the positive correlation between obese BMI and muscle GFAT activity is lost in insulin resistant diabetic individuals (57). At the cellular level, insulin appears to suppress the feeding associated potentiation of cytosolic *Gfat2* mRNA and protein in the liver of normal weight, standard chow fed mice (93). However, a similar elevation in ubiquitously expressed *Gfat1* was insulin insensitive. This, in conjunction with an increase in OGT-binding nuclear receptor LXRα, may have contributed to the selective elevation in nuclear *O*-GlcNAcylation seen between fasted and re-fed mice in this tissue (93). These adaptations likely facilitate OGT activation of transcription factors such as the lipogenic transactivators described in the previous sub-section. It should be noted, however, that the durations of fasting (24 hrs) and re-feeding (12 hrs) in this study were not equivalent to physiological timepoints (325). On the other hand, XBP1 splicing, and ER stress response that potentiates *Gfat1* transcription in cancer cells (61), does increase in mouse liver within a post-prandial timeframe [≤ 4 hrs post-meal (62)]. Importantly, induced XBP1s was also associated with a substantial increase in hepatic UDP-GlcNAc (62). In the fly body, *Gfat2* was found to be the more nutrient sensitive paralog. Feeding induced the transcription of *Gfat2*, with some concurrent inhibition of activity, but had little effect on *Gfat1* (64). Nevertheless, these relationships elucidate how post-prandial conditions potentiate and guide *O*-GlcNAcylation to promote the transcription and activity of proteins involved in the response to nutrient abundance.

By contrast, fasting is characterized by high FFA and low glucose availability with unique regulatory influences over HBP flux. Most models of nutrient deprivation are associated with a decrease in UDP-GlcNAc levels, even in models with simultaneous protein hyper-*O*-GlcNAcylation (37, 314, 315). These studies suggest that changes in OGT level, activity and substrate affinity are responsible, despite a loss of glucose driven HBP activity. Surprisingly, then, glucose and amino acid starvation stimulate XBP1s and GFAT1 levels *in vitro* (326) while nuclear receptor NR4A1, a known transcriptional enhancer of *Gfat2* (14, 327), is increased by fasting in rodent WAT (328) and liver (329). Higher GFAT availability could be a means to boost a localized and/or rapidly utilized supply of OGT substrate, even as the detection of overall UDP-GlcNAc levels remains low. Given that one endpoint of fasting stimulated *O*-GlcNAcylation is hepatic gluconeogenesis, which cannot run concurrently with glycolysis, it is feasible that lipid-directed F6P partitioning towards the HBP (see “UDP-GlcNAc Synthesis”) also facilitates targeted *O*-GlcNAcylation when extracellular glucose levels are low.

Outside the post-prandial period, the substrate specificity and outcome of *O*-GlcNAcylation is influenced by AMPK and p38 activation as well low insulin and dysregulated glucagon signaling. Fasting stimulated NR4A1 is downstream of CaMKII activation in the heart (327) and sensitive to hepatic glucagon signaling (329). These are interrelated processes in the liver wherein glucagon receptor activation induces a rise in intracellular Ca2^+^ that activates CaMKII [for review (330)]. This pathway was identified as the driving mechanism behind fasting *O*-GlcNAcylation of ULK1, which relied on CaMKII phosphorylation of OGT (316) but could also have turned on NR4A1 mediated *Gfat2* to enhance substrate supply. Furthermore, the outcome of ULK1 *O*-GlcNAcylation was to enhance its interaction with and activation by AMPK (316). This is conceptually in line with the observation that the *O*-GlcNAcylation of AMPK itself is stimulatory under pre-existing low glucose conditions. AMPK-dependent stimulation of OGT mRNA/protein has also been observed in glucose-deprived neuroblastoma cells (37). In that study, OGT substrate selection was also guided by increased interaction with p38 MAPK, which is activated in liver cells by both post-absorptive and prolonged fasting and by *in vitro* glucagon and LCFAs (331, 332). In addition to these OGT targeting mechanisms, low insulin signaling unmasks some *O*-GlcNAc regulatory relationships such as the potentiation of hepatic FoxO1 or the inhibition of PKA-dependent adipocyte PLIN1 activation, as previously discussed. While some of these mechanisms may be exclusive to starvation [e.g. XBP1s/GFAT1 (326)], others appear to be an early response to nutrient depression [e.g. p38 activation (37)] suggesting a role in physiological fasting as well. Regardless, these examples are merely a representative sampling of how feeding related shifts in intracellular conditions can alter the substrate-specific status and effect of *O*-GlcNAcylation.

Obesity is associated with systemic and intracellular metabolic changes that flatten the fed to fasting transition and its capacity to direct distinct *O*-GlcNAcylation patterns. The efficacy of insulin as a post-prandial signal is blunted due to high constitutive levels with depressed glucose-stimulated secretion plus multi-tissue insulin resistance. Hepatic *de novo* lipogenesis remains insulin sensitive, however, elevating its level of activation during the fasting period. Liver FFA content and a hyperglucagonemia (333) overdrive fasting gluconeogenesis while reduced insulin stimulation of post-prandial cellular uptake also diminishes glucose as a demarcation of recent consumption. Persistent hyperlipidemia, related to the disinhibition of adipocyte lipolysis/FFA leak (fed state) plus high VLDL production rate with delayed and less effective TAG clearance (fasting), drives ER stress and shifts the circulatory lipid signature throughout the day. This may be exacerbated inside cells by potentiated FFA but not LPL-dependent uptake as well as a reduced utilization of FAO and upregulated lipid storage mechanisms. Persistent nutrient excess, including HFD, depresses AMPK expression and activation [see (334)]. Consequently, nutrient and hormone responsive OGT targeting or interaction-based outcomes are likely to become less dynamic and coupled to eating in obese individuals. In addition, however, this scenario introduces mixed state regulatory forces on *O*-GlcNAcylation such as glucose-driven HBP activity with lipid F6P partitioning, glucagon stimulated OGT targeting without AMPK activation and the unmasking of insulin-inhibited substrates in a high UDP-GlcNAc/OGT environment. These combinations create the potential for novel and dysfunction-oriented *O*-GlcNAcylation patterns that are not seen in a physiological state.

Furthermore, proteins that target OGT and HBP flux are specifically dysregulated in response to obesity or HFD. Most of the proteins described above are elevated in the liver of obese humans and/or rodents including the protein level and activity of FAS (335–337) and LXRα (94–96). An increase in XBP1 splicing (338) and p38 activation (21, 339, 340) have also been reported. Contrastingly in adipose tissue, prolonged hyperlipidemia depresses FAS (337, 341, 342) and p38 transcription and activation [reviewed in (343)] while reports on LXRα have been mixed (338, 344, 345). In some cases, these tissue specific differences have been linked to a shift in lipid storage from adipose to liver in progressed obesity (337, 345). To that end, FAS and LXRα stimulate *de novo* lipogenesis in both tissues (346) while p38 alters hepatic FGF21 secretion to stimulate adipocyte FFA lipolysis (347). In the liver, it seems likely that the upregulation of these proteins contributes to obesity associated hepatic hyper-*O*-GlcNAcylation, particularly in the nucleus (21). However their role in shaping HFD WAT *O*-GlcNAcylation patterns is less clear. It is worth noting that NR4A1 gene expression is higher in obese human and rodent WAT (348, 349) while nutrient driven UDP-GlcNAc synthesis is expected to contribute in both tissues as well. One precaution to interpretation is that these proteins and transcripts were investigated exclusively in random-fed animals. Nevertheless, the sensitivity of their expression to *in vitro* hyperlipidemia and/or cellular stress, which are tonically elevated in obese tissues, suggests that the changes are likely constitutive and contribute to inappropriate regulation of *O*-GlcNAc-mediated cellular programming in both states. **Table 2** summarizes qualitative differences between the fasting and post-prandial milieu under physiological and obese conditions.

**Table 2:** Hepatic *O-*GlcNAc Interactome in Physiology and Obesity.

	Physiological		Progressed Obesity	
	*Fasting*	*Post-Prandial*	*Fasting*	*Post-Prandial*
Insulin	**↓**	**↑**	**↑**	**↑**
Glucose	**↓**	**↑**	**↑**	**↑**
ER Stress	**↓**	**↑**	**↑**	**↑**
Glucagon	**↑**	**↓**	**↑**	**↑**
FFAs	**↑**	**↓**	**↑**	**↑**
AMPK Activity	**↑↑**	**↓**	**↑**	**↓**
FAS (OGT)	**↓**	**↑**	**↑**	
LXRα (OGT)	**↓**	**↑**	**↑**	
XBP1s (*Gfat1*)	**↓**	**↑**	**↑**	
p38 (OGT)	**↑**	**↓**	**↑**	

A representative sample of circulating and intracellular conditions significant to the tone, targeting and outcome of O-GlcNAcylation is shown for a hypothetical liver cell. High serum insulin and glucose, as well as a mild ER stress, mark the post-prandial state while elevations in the glucagon, circulatory FFAs and AMPK activation characterize fasting in the liver. These ambient factors influence the availability of OGT substrates for and can alter the functional outcome of the O-GlcNAcylation. They also contribute to the expression and activation of proteins that potentiate OGT stability, activity (Fatty acid synthase, FAS) and nuclear localization (Liver X Receptor α, LXRα) and Gfat1 transcription (spliced XBP1, XBP1s) to elevate global but especially nuclear O-GlcNAcylation during high nutrient periods. Similarly, nutrient deprivation activates OGT binding p38 and post-translational modifier AMPK to guide fasting-appropriate substrate targeting despite a general decrease in O-GlcNAc tone. Obesity flattens these differences, driving up fasting insulin and glucose as well as post-prandial glucagon and FFAs. Relative nutrient excess stimulates ER stress and depresses AMPK activation. OGT and GFAT1 targeting proteins appear to be constitutively elevated. Under these conditions, which serve as representative examples, O-GlcNAc regulation of protein and transcriptional activity is less sensitive to context and more likely to re-enforce metabolic pathology. Arrows represent a qualitative assessment of relative differences in level or activity between the conditions – blue down arrow (lower), red up arrow (higher) with the number of arrows expressing magnitude. Under physiological conditions, there is a clear delineation between the fasting and post-prandial environment that is blunted in obesity.

## Summary and future directions

Despite its traditional classification as a glucose metabolism associated modification, the *O-*GlcNAc regulatory system interacts extensively with lipids and appears central to support lipid homeostasis. Hyperlipidemia (e.g. *in vitro* fatty acid exposure, diet induced obesity) potentiates protein *O-*GlcNAcylation in many cell types including gastrointestinal tissue, adipose, muscle, heart and liver. A biphasic response was noted in pancreatic β-cells and macrophages with an early rise in *O-*GlcNAcylation followed by a drop below baseline in models of more progressed obesity. In some neural cells (i.e. hippocampus, neuroblastoma cells), elevated lipid exposure depressed *O*-GlcNAcylation under all tested conditions. It is not clear whether these examples of lipid stimulated hypo-*O*-GlcNAcylation are exclusive to the noted tissue types or if the available data is restricted by experimental variability. More consistent standards in the use of subject sex and species, lipid type and duration, endpoint measurements and the use of multi-timepoint assessment would significantly aid in understanding this complex physiology.

Cellular *O*-GlcNAcylation patterns are commonly associated with changes in the enzymes that direct HBP UDP-GlcNAc synthesis and with the glycosylation enzymes OGT and OGA. However the relative contributions of each were context dependent. Mechanisms linking lipids to the HBP rate-limiting enzyme GFAT include transcriptional potentiation by ER stress protein Xbp1s and lipotoxicity sensor NR4A1 as well as inhibitory AMPK phosphorylation. Despite serving the same HBP function, the paralogous GFAT1 and GFAT2 proteins, which are not always experimentally distinguished, appear to be independently regulated and show distinct responses to ingested nutrients and different lipid species. Moreover, most of the enzymes that regulate glycolysis and the HBP are fat sensitive, particularly around the nexus of F6P metabolism. The effects of LCFA or LCFA-CoAs on GFAT/GNPDA and PFK/FBPase all appear to direct glucose towards the HBP at the expense of glycolysis under hyper-*O*-GlcNAcylating conditions. Numerous lipid-sensitive OGT modification strategies have been documented including transcription changes, post-transcriptional suppression by micro-RNAs, FAS-potentiated protein stabilization and activity, AMPK-dependent substrate preference and shifts in subcellular localization to bind nuclear receptor LXR or membrane-bound PIP3. OGA regulation has not been deeply characterized in this context but FAS depresses its activity and a mitochondria/lipid droplet associated splice isoform, sOGA, has been identified. Thus, the *O*-GlcNAc regulatory system evinces a surprising flexibility in lipid response strategies despite its limited diversity of direct enzyme regulators.

In addition to being lipid responsive, the *O-*GlcNAc regulatory system plays a significant role in lipid physiology, including dietary fat intake. Systemic lipid uptake is determined by the overall amount of food consumed and the preference and intestinal absorption of its fat content. Under physiological conditions, hypothalamic *O-*GlcNAcylation is increased by fasting in orexigenic AgRP neurons and by feeding in anorectic ARC and PVN neurons. In both cases, OGT activity is required to maintain normal excitatory activity and/or neuronal viability, as well as AgRP ghrelin sensitivity and ARC leptin sensitivity. In addition, OGT acts outside the CNS to support the synthesis and HFD upregulation of satiety hormones, including adipocyte-secreted leptin and β-cell insulin. These findings implicate *O*-GlcNAcylation in the homeostatic regulation of eating but OGT also influences dietary fat preference specifically. Adipocyte OGT is required for transient HFD hyperphagia by coupling lipid level to the secretion of hunger-promoting endocannabinoid hormone AEA. Macrophage OGT depletion also potentiates HFD overconsumption, possibly downstream of inflammation-related taste bud remodeling or insulin resistance in mesolimbic dopamine neurons that control the hedonic motivation to eat. In macrophages, OGT is a downstream inhibitor of TLR4 signaling, which is also necessary for dietary sugar and fat preference suggesting that its interactions with OGT in mesolimbic cells and on the tongue merit further investigation. *O*-GlcNAcylation in the intestine may support dietary lipid extraction by limiting local GLP-1 secretion, which otherwise suppresses enterocyte chylomicron-TAG output. Furthermore, *O*-GlcNAcylation *in vitro* upregulates two important lipid sensing proteins - PLIN2 and CD36. LD membrane protein PLIN2 facilitates cytosolic LD storage in multiple cells, including as a means to potentiate enterocyte lipid extraction. CD36 is similarly involved in multi-cellular FFA uptake and signal transduction that contributes to lingual fat sensitivity and enterocyte synthesis of chylomicrons and anorectic hormone OEA. Importantly, *O*-GlcNAcylation has not been phenotypically characterized in fasting-stimulated hunger or intestinal lipid absorption but has been directly tied to meal-related satiety and fat preference.

Context-sensitive OGT activity supports both pro-consumptive and negative feedback mechanisms on eating and lipid uptake emphasizing its role in homeostatic balancing. By contrast, hyperlipidemia-driven *O*-GlcNAcylation patterns show the potential to exacerbate obesity endophenotypes that drive pathological overeating. These include the uncoupling of orexigenic and anorectic signals from their nutrient context, an overall decrease in satiety and tonic elevations in the hedonic value and lipid extraction of food. Towards that end, adipocyte hyper-*O-*GlcNAcylation in obesity is consistent with the progressive increase in plasma AEA that supports food craving and reward. The opposite condition in macrophages, hypo*-O-*GlcNAcylation, is linked to proinflammatory signaling known to facilitate ghrelin, leptin and insulin resistance that numbs homoeostatic circuitry to nutrient status signals and feedback triggers. This is likely accelerated by *O*-GlcNAc stabilization of PTP1B and STAT3, proteins with a primary role in insulin and leptin resistance in the brain and peripheral tissues. Shifts in oxidative capacity or fuel preference, seen systemically in both OGA and OGT depressed animals, are likely to disrupt direct nutrient sensing in AgRP (FAO) and/or POMC (glucose oxidizing) neurons, consistent with the nutrient insensitivity of these regions in obese individuals. Unfortunately, *O-*GlcNAc responses to hyperlipidemia and obesity in most areas of the brain are not known so the relevant mechanisms in this state remain speculative.

Beyond ingestion, dynamic *O-*GlcNAcylation in peripheral cell types has been implicated in lipid uptake and utilization as well as the balance between nutrient storage and release. OGT activity stimulates CD36 dependent FFA uptake in heart cells and likely in adipocytes by potentiating LPL secretion, which is required for circulatory TAG hydrolysis. While these proteins have similar roles in other tissues (e.g. liver, skeletal muscle), their *O*-GlcNAc regulation has not been tested in that context. In addition to LPL, however, adipocyte *O*-GlcNAcylation tunes lipolytic capacity by inhibiting the PKA (i.e. fasting) phosphorylation of LD protein and lipolysis gatekeeper PLIN1 and possibly by mediating its membrane expression during LD maturation. In addition, dynamic OGT/OGA activity are required for the terminal differentiation phase of adipogenesis. This is likely related to *O*-GlcNAc’s inhibition of C/EBPβ and its potentiation of PPARɣ2, the latter of which drives lipogenic and adipokinic gene expression. Thus, the *O*-GlcNAc regulatory system contributes to the maximal lipid storage capacity of WAT and its metabolic flexibility to switch between post-prandial FFA uptake and fasting FFA release. The liver also takes on dual roles in lipid storage and release as well as fasting glucose production. Hepatic OGT potentiates the meal related activation of lipogenic enzymes (FAS, ACC) and transcription factors (ChREBP, LXR, SREBP-1c, FXR). However during fasting, hepatic AMPK and glucagon signaling steer *O*-GlcNAcylation onto select protein targets that activate gluconeogenesis, autophagy and FAO. Proper WAT and liver function, therefore, rely on differential detection of and regulation by the post-prandial and fasting states including relative glucose/FFA abundance, insulin/glucagon signaling and ER stress/AMPK activation. The *O*-GlcNAc regulatory system is similarly sensitive to these conditions, leading to unique variations in HBP flux, OGT behavior and substrate interactions that guide its context-dependent support for nutrient homeostasis in these tissues.

During early exposure, lipid-responsive *O-*GlcNAcylation in peripheral cells helps to accommodate dietary fat overconsumption by upregulating the mechanisms behind circulatory TAG clearance, cardiac FAO supply, hepatic nutrient buffering and WAT lipid storage. In the face of persistent hyperlipidemia, however, these same changes are counter-productive to cellular health due to excessive lipid accumulation, exacerbated by overactive hepatic lipogenesis and impaired adipocyte lipolysis and differentiation. Insulin resistance, facilitated by OGT depleted pro-inflammatory macrophages and adipose/liver hyper-*O*-GlcNAcylation, further impairs adipocyte FFA uptake and disinhibits FFA/VLDL secretion, adding back to circulatory hyperlipidemia. The imbalance of nutrient and hormone signaling blunts the fed to fasting transition, which feeds back onto the *O*-GlcNAc regulatory system itself. An oversupply of OGT, GFAT and HBP nutrient input together with context-insensitive OGT targeting and substrate access could fundamentally change the functional impact of *O*-GlcNAcylation and divorce it from sources of negative feedback. While not proven, this hypothesis suggests how the *O*-GlcNAc regulatory system, with its sole pair of enzyme controllers and a nutrient-promiscuous source of effector, can nevertheless diversely contribute to complex homeostatic relationships. Furthermore, it adds nuance to the “goldilocks zone” theory for how the same directional change in *O*-GlcNAc tone can be both beneficial and harmful under different circumstances.

In conclusion, *O-*GlcNAcylation is best understood as a multi-nutrient sensitive modification that has distinct glucose and lipid sensitive response profiles as well as a flexible array of outcomes dependent on cellular context. Under physiological conditions, cell type specific *O-*GlcNAcylation patterns contribute to low and high nutrient-cued homeostasis to influence the balance between hunger vs. satiety, fat preference vs. overconsumption, lipid utilization vs. storage vs. release. It may also serve as a form of intermediate length metabolic memory to tie recent or expected nutrient availability with markers of energy supply, demand and cell stress in guiding cellular adaptation strategies to acute nutrient conditions. Prolonged hyperlipidemia, however, appears to disrupt this carefully balanced system and shift the net outcome of *O-*GlcNAc regulatory activity towards obesogenic and metabolic dysfunction. Future investigations into the source of this shift - whether differences in *O-*GlcNAc levels, OGT/OGA targeting, or the larger *O-*GlcNAc interactome – may prove fruitful in delineating and abrogating the self-sustaining pathways between progressive obesity and metabolic disease.

## Author contributions

AL conceived the initial review topic and created the first draft of the manuscript and figures. AL and JH contributed together to the ongoing conceptual development and revision of the final manuscript and figures.

## Funding

Funding was provided by NIDDK intramural research program grant ZIADK060103.

## Conflict of interest

The authors declare that the research was conducted in the absence of any commercial or financial relationships that could be construed as a potential conflict of interest.

## Publisher’s note

All claims expressed in this article are solely those of the authors and do not necessarily represent those of their affiliated organizations, or those of the publisher, the editors and the reviewers. Any product that may be evaluated in this article, or claim that may be made by its manufacturer, is not guaranteed or endorsed by the publisher.
